# Supplementary material for: Socioeconomic disparities in mobility behavior during the COVID-19 pandemic in developing countries
Source: EPJ Data Sci. 2025 Mar 24;14(1):25. doi: 10.1140/epjds/s13688-025-00532-2 (PMC11933202; doi:10.1140/epjds/s13688-025-00532-2)
Supplement: Supplementary file 1 — (PDF 6.5 MB) [file 13688_2025_532_MOESM1_ESM.pdf]

Socioeconomic disparities in mobility behavior during the COVID-19 pandemic in developing countries

Lorenzo Lucchini,[1,2,3,4] Ollin D. Langle-Chimal,[3,5] Lorenzo Candeago,[3] Lucio Melito,[3] Alex Chunet,[3] Aleister Montfort,[3] Bruno Lepri,[4] Nancy Lozano Gracia,[3] and Samuel Fraiberger[3]

[1]*Centre for Social Dynamics and Public Policy, Bocconi University, Milan 20100, Italy*

[2]*Institute for Data Science and Analytics, Bocconi University, Milan 20100, Italy*

[3]*World Bank, Washington - DC*

[4]*Fondazione Bruno Kessler, Trento - Italy*

[5]*University of Vermont, Burlington - VT*

SUPPLEMENTARY INFORMATION

SI 1. GPS SOURCED MOBILITY DATA

This section provides supplementary information about the data used in our analyses. The data provider, Veraset, is an SDK aggregator working all around the globe, collecting GPS mobility traces from personal devices of individuals who opted into their services.

Veraset's Movement dataset comprises raw, pseudonymous GPS data collected from a diverse array of mobile applications and through several Software Development Kits (SDKs) and aggregators [17, 18]. This approach enables extensive coverage of mobile phone location information across various countries. By integrating data from multiple sources, Veraset ensures a comprehensive representation of human mobility patterns. Veraset aggregates location data from numerous apps and SDK providers, enhancing the dataset's comprehensiveness and reducing reliance on a single data source. The collected data undergoes deduplication, validation, cleansing, and standardization to ensure quality and readiness for analysis.

In general, GPS data collection via mobile devices can introduce biases such as sampling bias (i.e., the dataset may not be representative of the entire population), and spatial biases (e.g. urban areas might be overrepresented due to higher smartphone penetration and app usage compared to rural regions) [11]. This work partially overcomes these issues by i) pooling GPS data from different sources (different SDK providers and apps), and ii) by employing population reweighting techniques to recover local area's population representativity (see Methods). In our analysis, we focus on middle-income countries from different geographical areas.

SI 2. USER DISTRIBUTION

We select 6 middle-income countries and follow individuals' trajectories over a period of almost one year (2020). The selected countries are Brazil, Colombia, Indonesia, Mexico, the Philippines, and South Africa. The largest bulk of these users lies in the urban/metropolitan areas of the main cities of each country. Figure SI 1 shows the geographical distribution of the GPS points registered from the users' personal devices.

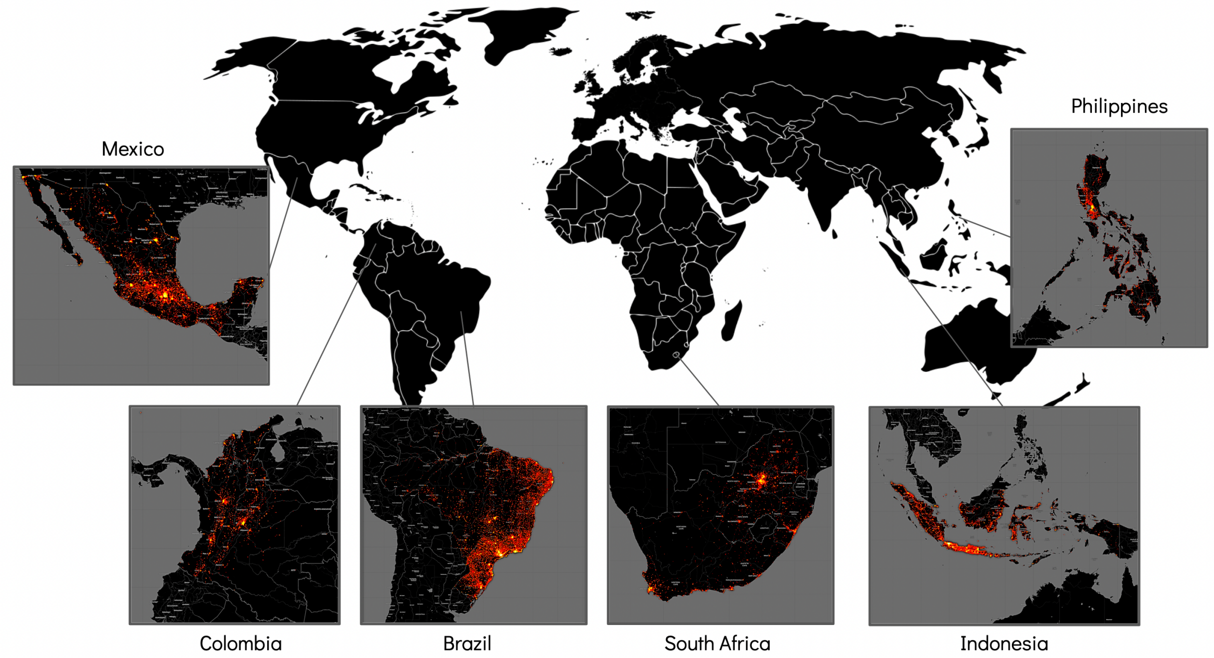

Figure SI 1. User distribution across the 6 countries.

SI 3. MOBILITY DATA PROCESSING DETAILS

A. From pings to stop events

The first step aims at aggregating pings into consecutive groups of pings called "stop events". To this end, previous work [2, 9] has shown how starting from GPS trajectories, it is possible to aggregate the information in terms of a series of stops an individual performed. In this perspective, stop events filter inaccurate GPS points and discard non-stationary events from the series [1, 9].

Schematically, in this first step, the following actions are performed:

1. A rough filter on accuracy is performed to exclude pings with inconsistent or too large accuracy values, i.e. $accuracy \geq 0m$ and $accuracy \leq 200m$.

2. A second filter is performed by checking coordinates values and constraining them to the latitude and longitude domain, i.e. $-90 \leq lat \leq 90$ and $-180 \leq lon \leq 180$.

3. Third, we only kept pings that were timestamped within the temporal period of days Veraset provided. Pings with timestamps outside the temporal window are potentially introduced by delays in the upload process.

4. We then discarded all users who were not significantly active during the validation period.

We then define a "stop event" as a consecutive sequence of pings for which the distance between each pair of pings is smaller than $r = 25m$. We additionally require that the maximum time between two consecutive pings of the sequence should be less than 1 hour for the sequence to be considered part of the same stop event. Similarly, we fix the minimum distance between the earliest time of a candidate stop event and the latest to be at least 5 minutes. To each stop event, we associate a *lat-lon* tuple based on the medoid of the latitude and longitude of the pings forming the stop event. After this processing, stop events with an average pings' accuracy greater than $100m$ are discarded as an additional quality requirement.

To each stop event is then associated a unique "GEOMID", representing a unique administrative unit in the *administrative boundaries dataset* using the *H3 library* [16] and *Apache Sedona* [19].

B. From stop events to stop locations

This second step applies a clustering algorithm (we adopted the DBSCAN algorithm [7]) to all the stop events of a single individual. We used a value of the ϵ parameter (*epsilon*), as defined in the Python *sklearn.cluster.DBSCAN* class [7], that corresponds to a distance between stop events of 25 meters. This results in an assignment to each stop event of a label identifying all other stop events clustered together by the algorithm. We call the label of each stop event "stop location".

C. From stop locations to home, work, and other locations

Reliable home and work identification algorithms are essential to map an individual's mobile phone data-trace to their socioeconomic background. During the past decades, several efforts have been made to correctly assign such labels to a coordinate pair for a single user [1, 5, 6, 12]. In this work, we build upon the preferential return mechanism based on the exploration/exploitation dichotomy [3, 13] segmenting by important heuristics such as the day of the week and visiting hours.

Given that we are interested in changes in the users' primary homes, we propose a pipeline to define and optimize the labeling of a given cluster or stop location as a possible home or work defined dynamically. Intuitively, the algorithm runs for each user by rolling over windows built of a definite number of days to infer during each period which location (among those visited within the window and provided as input) is the users' home and which are their work locations (multiple work locations are allowed). In order to do this, we define thresholds with the minimum amount of days when the cluster is visited (*min_periods_over_window*) over a rolling window of days (*period_window*). We use two different sets of these parameters to define

both home and work. The difference between the labels is given by the heuristics of plausible times when a user might be present at that given location.

With this in mind, we first define nighttime as the period of hours comprised by the time between 11 pm and 5 am of the following day. The rest of the day is marked as daytime. We also consider a working day to be Monday to Friday while leaving Saturday and Sunday as weekends. Then, we define as home candidates those stop clusters that are visited on weekends or during "nighttime" and as work candidates those visited during the complementary hours of the week. We add an extra restriction to work by setting a minimum amount of average time per day spent there. When the candidates are computed all the "work" labels are kept while only the most frequently visited "home" within the window is considered as such. The remaining clusters are then labeled as "other". Labels are assigned dynamically, meaning that, in each time window, we look at a user's home location and work locations independently. However, if a stop location is labeled as either a home or work location at least once, i.e. in at least one-time window, we expand the labeling to all visits ever performed by the user to that location, since it is likely to be always related to residence or work activity conducted before or after it's classification.

D. Activity filter

Following the same perspective, aiming at providing a reliable and long-term dataset of individuals to which a socioeconomic status can be assigned, additional filters on the minimum level of activity for each individual are applied. The idea is to perform the analysis on a set of individuals who remain active up to a minimum level, to ensure a minimum longitudinal coverage and thus ensure a sufficient level of significance for the computed metrics. More specifically, these filters require i) a minimum number of days of activity before the beginning of March 2020, and ii) a minimum level of activity over the entire observation period. Here an individual is defined to be active on a day if she/he has at least one stop event recorded on that day. The "minimum level" of activity is defined as a 20% day threshold over each of the two periods separately.

For the specific case of the Republic of Indonesia, the period before the pandemic started at the beginning of February 2020 to avoid the inclusion of individuals who were later pulled out due to a major customer dropout. The "during pandemic" period remains unchanged.

SI 4. EXPANDING POLYGONS AND FILLING GAPS BETWEEN SMALLER ADMINISTRATIVE UNITS

High-precision measurements of mobility patterns are difficult to connect to equally precise and fine-grained demographic data. Our approach, as described in Material and Methods (section "Administrative boundaries data"), leverages over local administrative data at the smaller administrative units for each country. Often, these data consist of polygons and summary demographics for the area contained within each polygon. At the finest level these polygons, i.e. administrative units' boundaries, consist of blocks and/or neighborhoods within cities. We take advantage of the geospatial data distributed by the statistical agencies of each country, which makes it difficult to homologate the polygons at the sub-national level. The highly precise registration of administrative polygons often neglects to include local streets and other neighboring elements of the city in favor of a more accurate representation of blocks and places of interest such as parks. Albeit being these polygons precise from an administrative perspective, they result in an incomplete coverage of the city area. In the interest of connecting stop locations and demographic data, the gaps between them are responsible for a partial spillover of the mobility data at our disposal. Being a cluster location an object consisting of several stops happening at different times but in a restricted radius area, they are sensitive to boundary effects ultimately induced by both the limited accuracy of GPS locations and the stop and clustering processing. To reduce this loss of data, i.e. to assign an administrative unit also to cluster locations that fall outside high-precision administrative borders, we expand these borders filling the gap areas with the following approach. For each country, we first identify all the polygons surrounded by empty space (i.e., those are the only ones to be expanded), and those which are not. We merge all the polygons without any gaps between them to create a country mask. Then, we repopulate the polygons to expand by interpolating new spatial points every 2 meters; this step yields the same polygons but with denser borders defining them. We use this each of these spatial points as centroids to generate a Voronoi tessellation using the mask from the first step [4]. Finally, we find all the Voronoi polygons that touch an original one and redefine the extent of the latter by the union of the former ones.

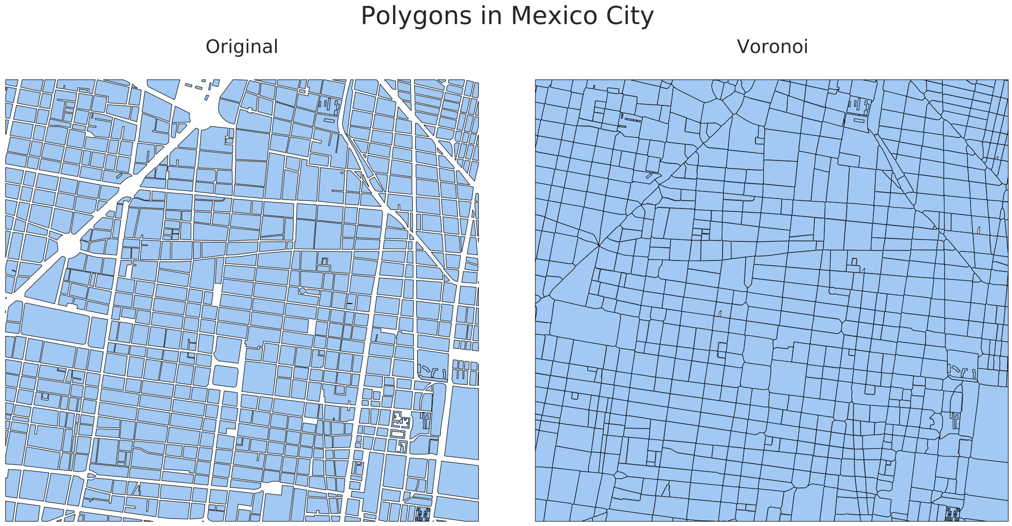

Figure SI 2. Polygon expansion of administrative units in urban areas.

SI 5. VALIDATION OF HOME/WORK STOP LOCATIONS PATTERNS

Particular care was devoted in this work to the classification of stop locations into “home” and “work” locations. To produce reliable labels, a subset of devices’ trajectories was independently manually annotated. Two independent annotators were asked to look at all the stop locations of 500 different users across five different countries and label them as either “home”, “work” or “other” locations. Annotators were provided with information about nearby points of interest, satellite imagery, and summary statistics about each stop location. Point Of Interest (from both OpenStreetMap and Google Maps) and satellite imagery (from Google Maps) were provided in the form of an explorable map. Summary statistics informed annotators about the fraction of time spent in a location daily spanning over the entire study period, the fraction of time spent on different weekdays over the average week (to provide insights on weekday-weekend patterns), and the fraction of time spent over different hours of a day for an average day of the year.

The resulting labels of the two annotators were then compared and, when different opinions were expressed, a third independent annotator was asked to tie-break selecting the best label, in their independent opinion.

Performances were tested against the annotated labels to see how well the labeling algorithms were performing. Grid search exploration was used to check for different algorithm configurations, tuning five different parameters: time-window size for home location detection, time-window size for work location detection (*period_window*), the minimum number of days an individual visited a stop location within the period window for both home and work location separately (*min periods over window*), and the minimum

frequency of time an individual should spend at their workplaces to label those locations as work locations. Performances were tested using:

- Cohen's Kappa test: reaching statistically comparable results, in terms of the agreement between the two independent annotators and the tie-breaker annotator's labels and the algorithm ($K = 0.93 \pm 0.03$ and $K = 0.93 \pm 0.02$ respectively)
- macro averaged Accuracy: $A = 0.80 \pm 0.03$
- macro averaged F1-score: $F1 = 0.87 \pm 0.02$

Errors were estimated through bootstrapping with a 100 independent sample of 500 users' stop locations with replacement. Noticeably, the algorithm was particularly reliable in inferring home locations, scoring an average cross-country F1 of 0.94 ± 0.02.

SI 6. DISTRIBUTION OF TIME SPENT

We find that for all of the studied countries, the time spent at home locations was increased relative to a pre-pandemic baseline. This increment spans across different wealth groups. The location with the largest time spent reduction was the individual's workplace. Interestingly, the high-wealth users increased their time spent at home more than their low-wealth counterparts while they also reduced their time spent at work. In general, the share of time spent at third locations was also reduced.

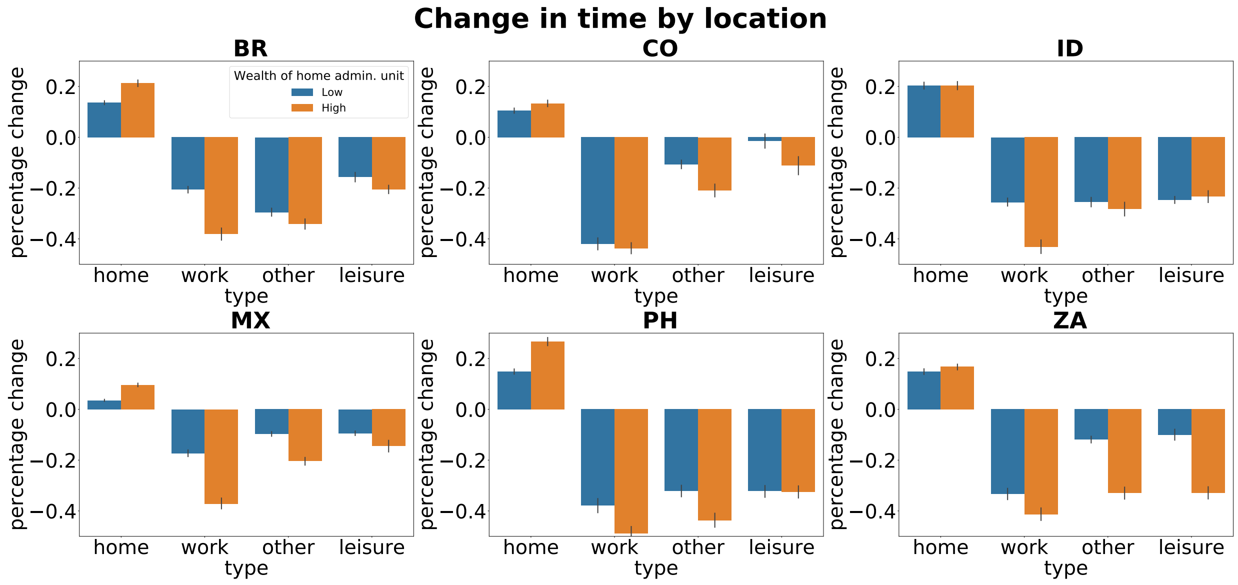

Figure SI 3. Percentage change of time spent by location.

SI 7. RESULTS BY CITY

In the main paper, we show the aggregated results of users with the primary home location assigned to any of the 5 largest cities of each country. Figures SI 4 and SI 5 show the share of users not leaving their homes and the share of users commuting to work only in the largest city of each country.

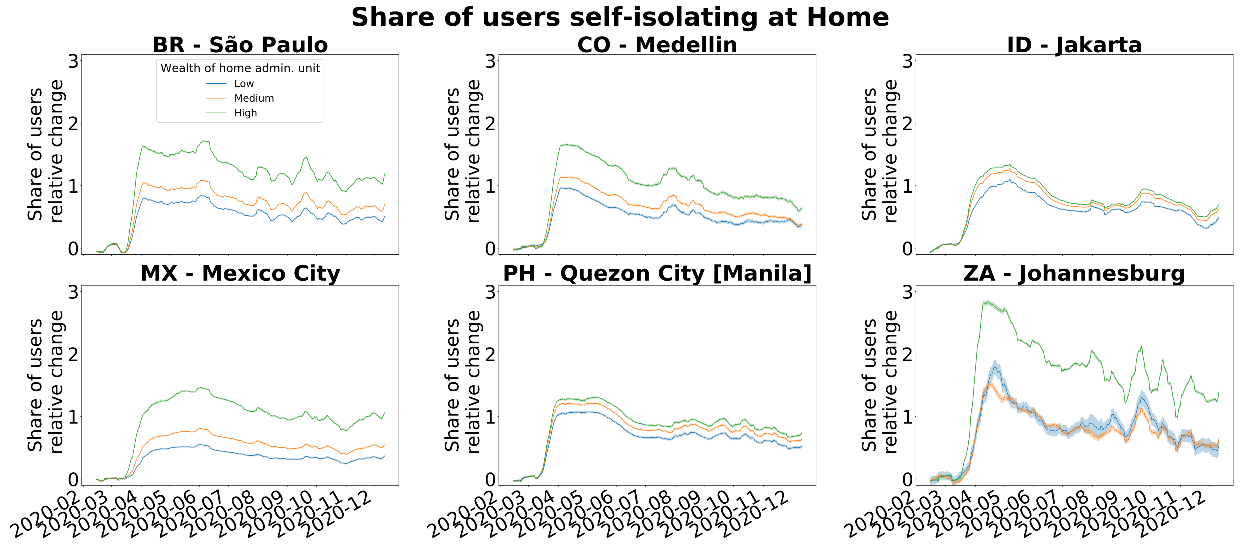

Figure SI 4. Percentage change of share of users isolating at home by wealth of administrative home unit in the largest city (by coverage).

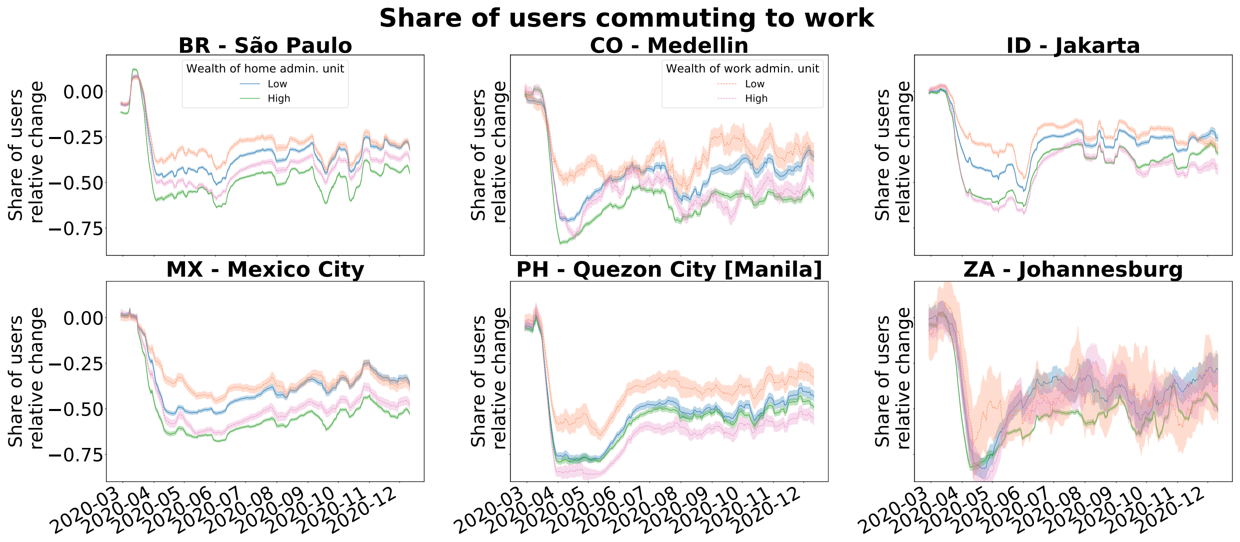

Figure SI 5. Percentage change of share of commuters by the wealth of administrative home unit and low wealth commuters by wealth of administrative work unit in the largest city (by coverage).

SI 8. GAPS BETWEEN HIGH AND LOW INCOME GROUPS

This section illustrates directly the gaps between income groups over the entire period of our analysis.

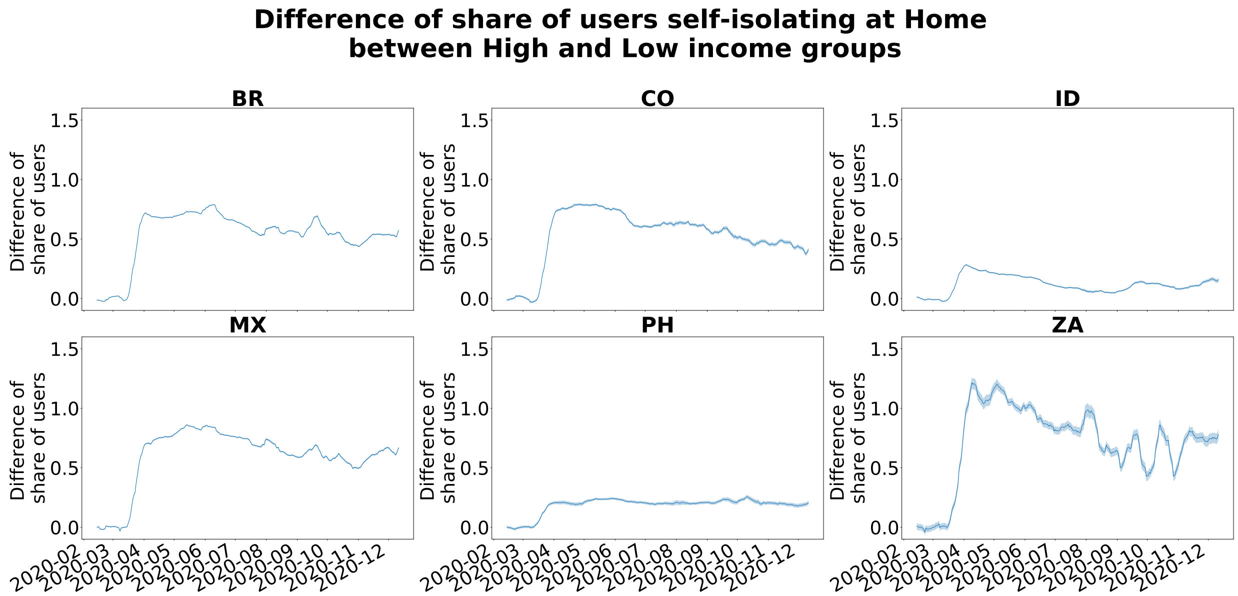

Figure SI 6. Difference of the percentage change of share of users self-isolating at home by wealth of administrative home unit in the whole country.

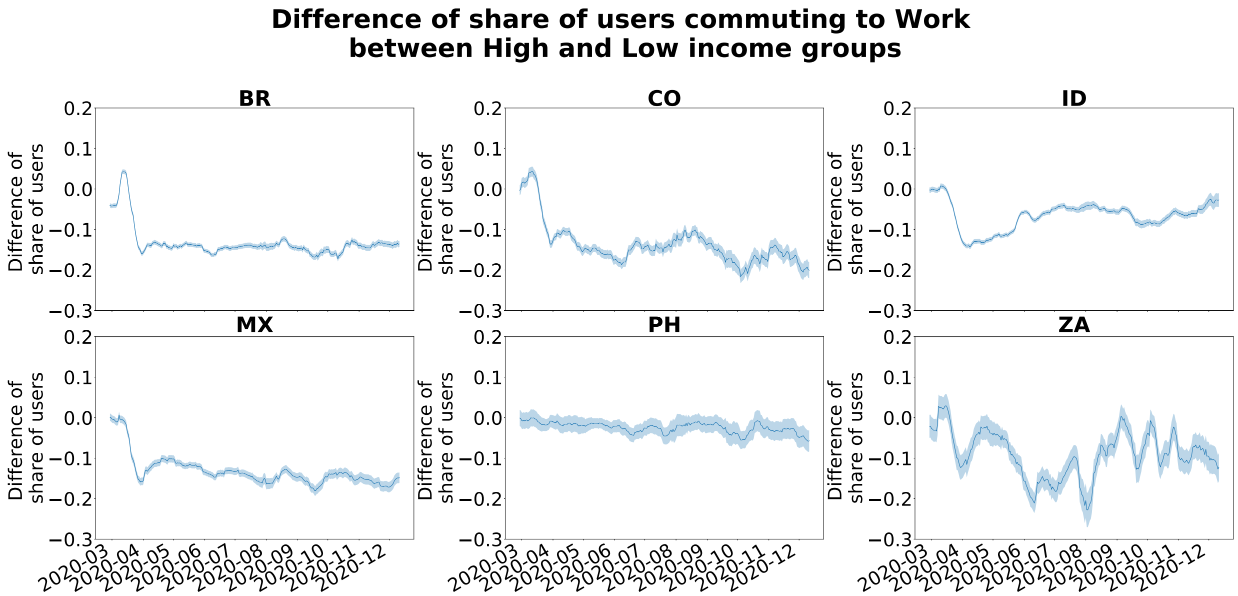

Figure SI 7. Difference of the percentage change of share of commuters by wealth of administrative home unit in the whole country.

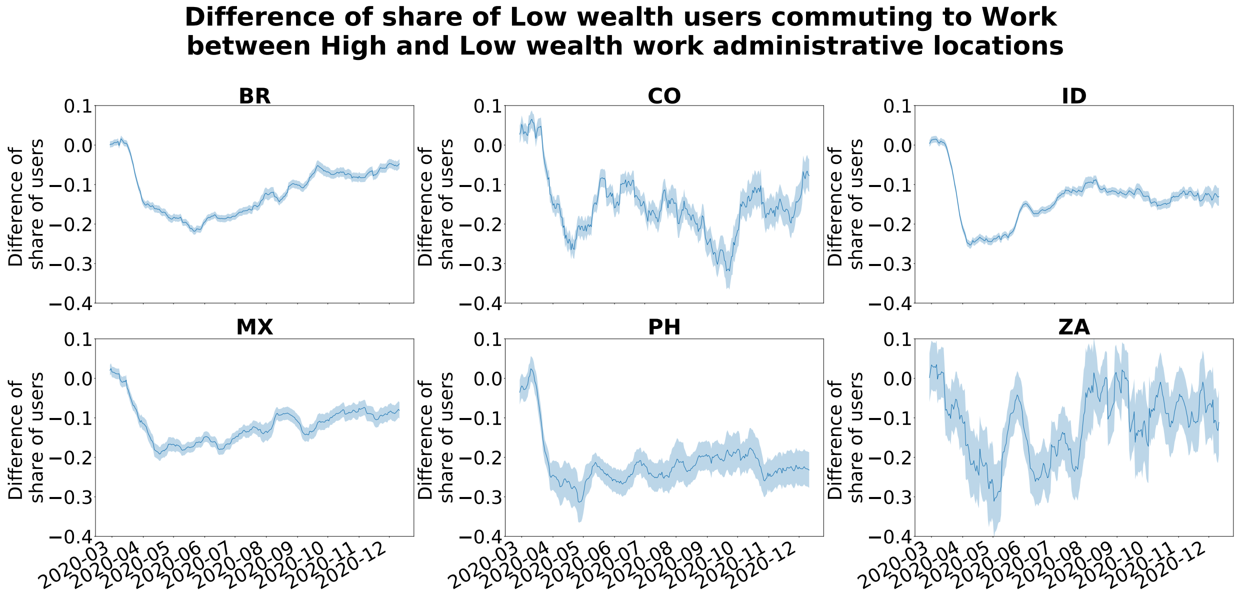

Figure SI 8. Difference of the percentage change of share of low wealth commuters by wealth of administrative work unit in the whole country.

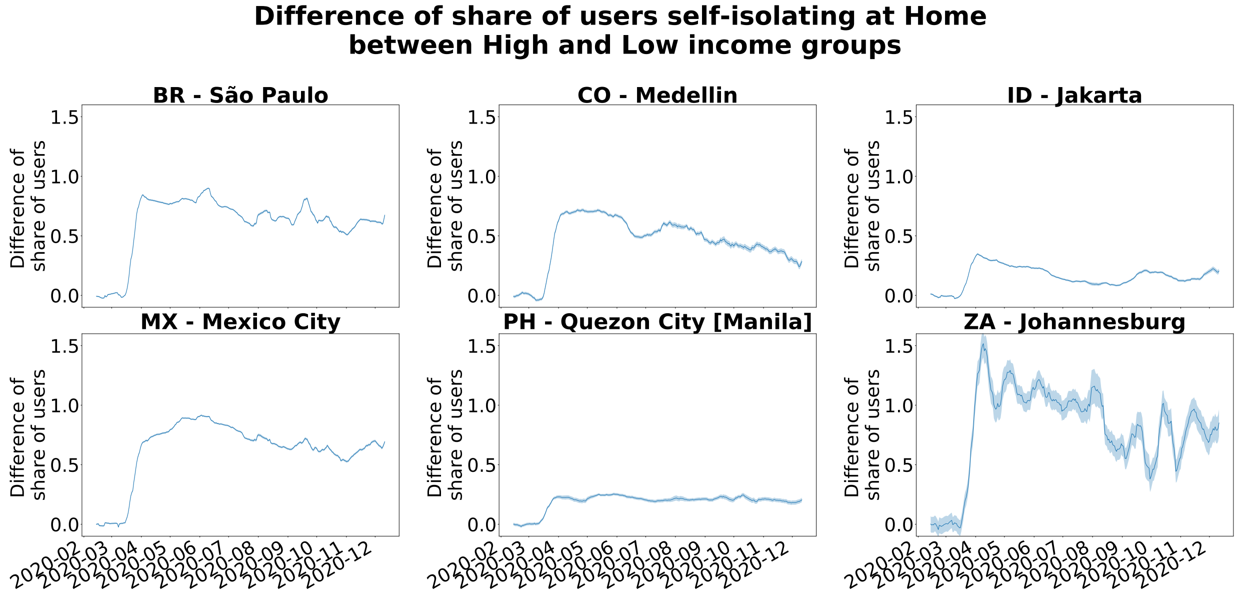

Figure SI 9. Difference of the percentage change of share of users self-isolating at home by wealth of administrative home unit in the largest city (by coverage).

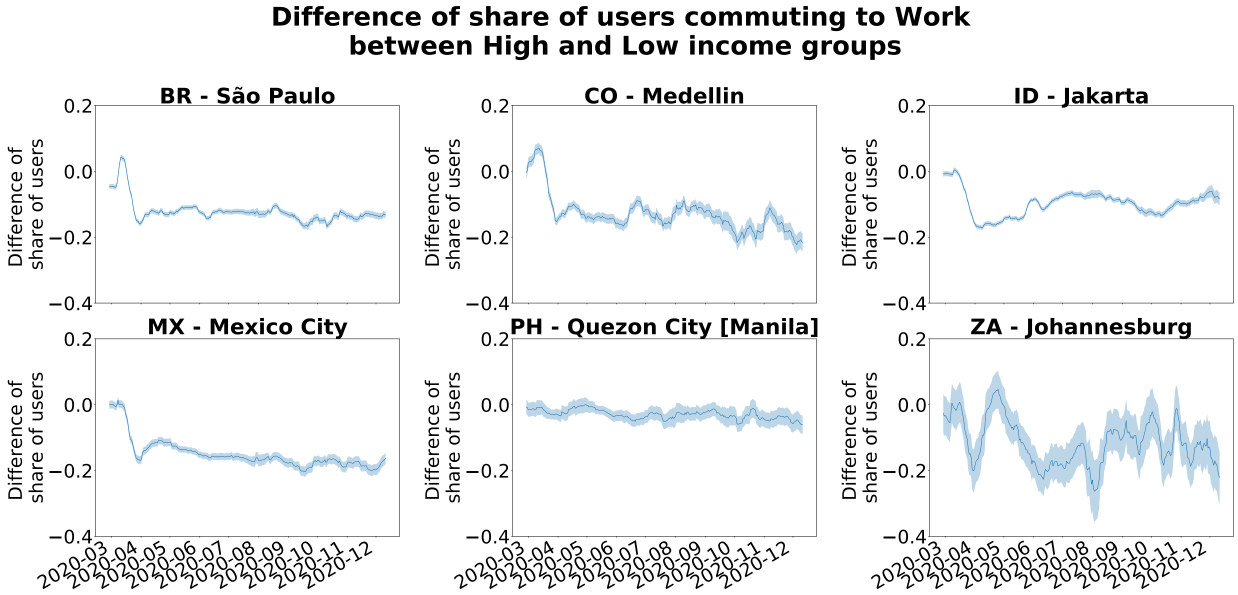

Figure SI 10. Difference of the percentage change of share of commuters by wealth of administrative home unit in the largest city (by coverage).

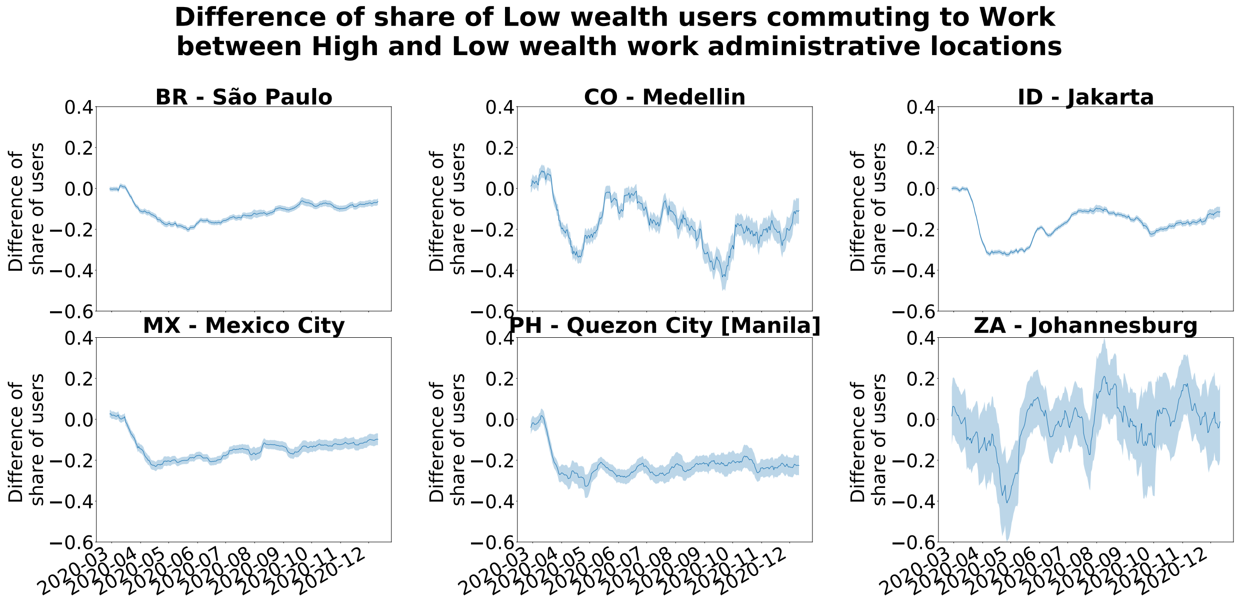

Figure SI 11. Difference of the percentage change of share of low wealth commuters by the wealth of administrative work unit in the largest city (by coverage).

SI 9. MIGRATION PATTERNS

In the main text, we discuss and present migration patterns from urban to rural areas. Here we show complementary migration patterns: cumulative migration curves from urban to rural (see Fig. SI 12), from rural to urban (see Fig. SI 13), and the net daily share of users migrating from urban to rural areas (see Fig. SI 14).

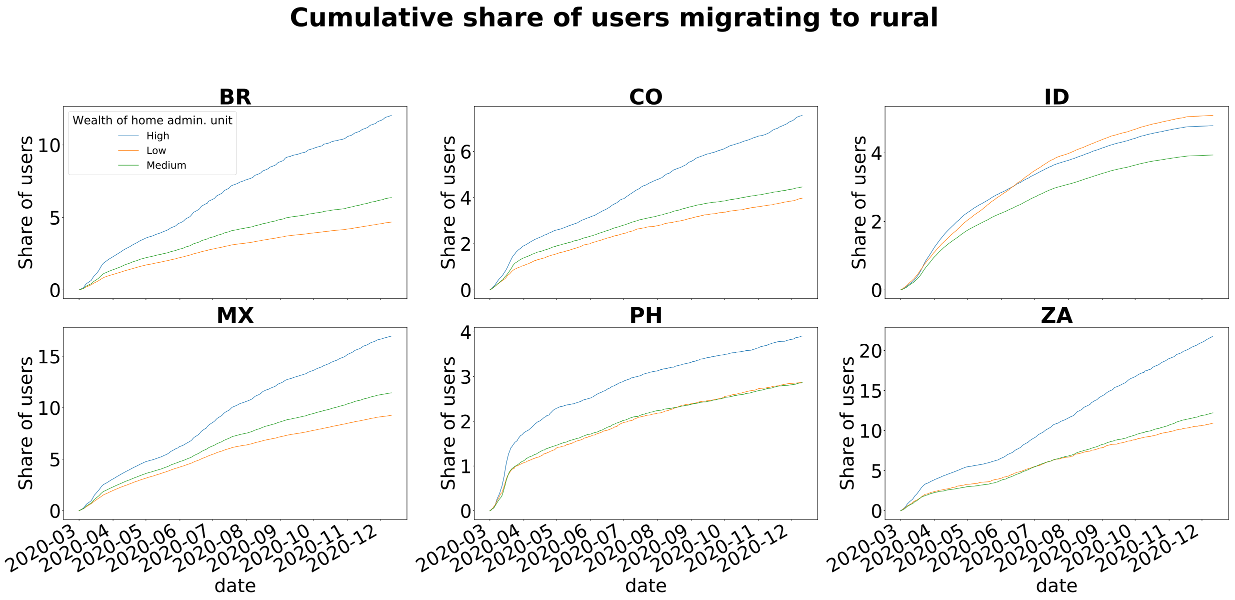

Figure SI 12. Cumulative share of users migrating from urban to rural areas.

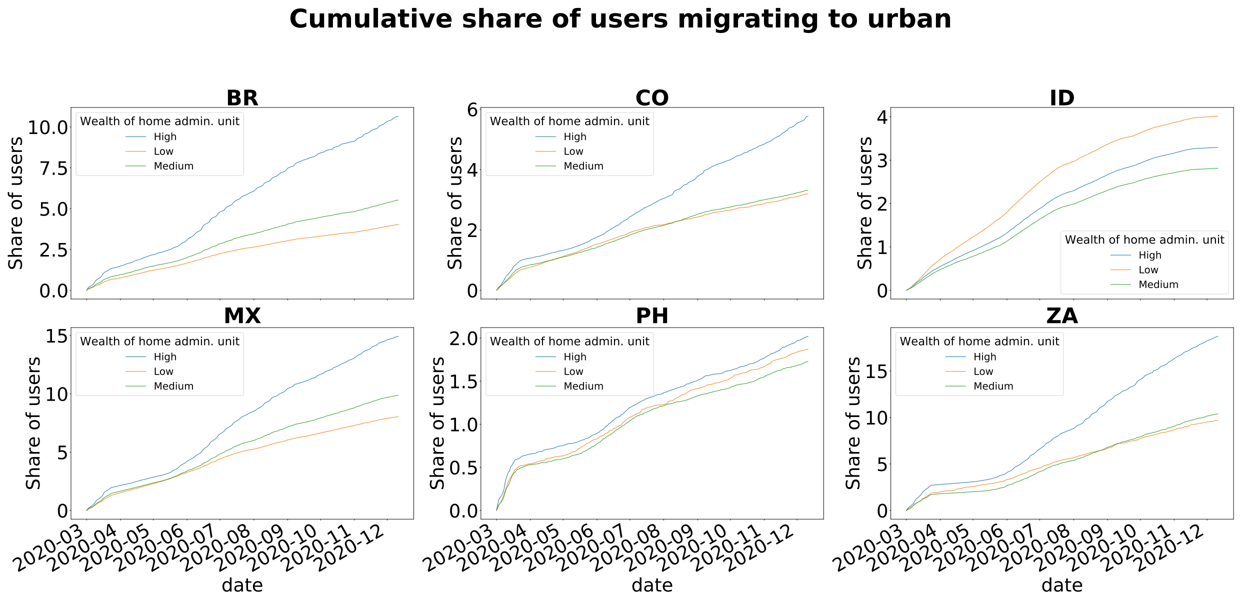

Figure SI 13. Cumulative share of users migrating from rural to urban areas.

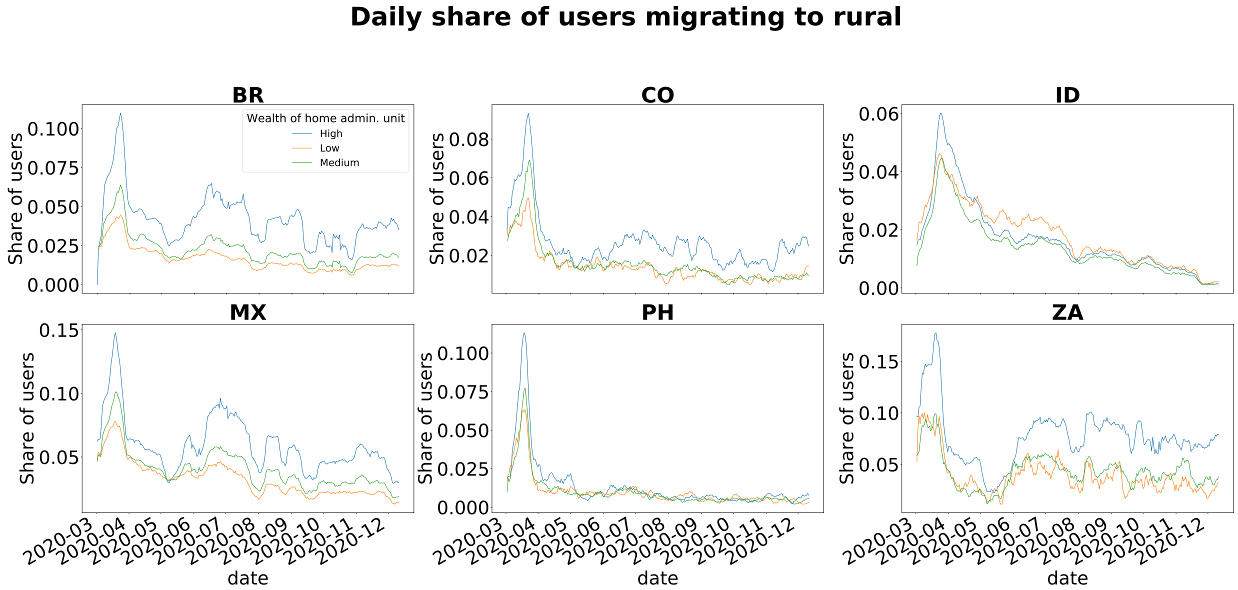

Figure SI 14. Daily share of users migrating from urban to rural areas.

SI 10. COMMUTING PATTERNS BY WEALTH OF RESIDENTIAL AREA

In the main text, we discuss and present mobility behavior through commuting patterns interruptions. This section focuses on all the population groups as in the main manuscript, providing additional insights, for each wealth group separately, on the specific changes in commuting behavior based on the wealth of the workplace. In the main text, we reported the change in the fraction of individuals who are not commuting from home to work focusing on the behavior of individuals living in "low-wealth" neighborhoods. This section complement those findings including results also for individuals living in "medium-wealth" and "high-wealth" neighborhoods. The three figures together show that the disparity between individual working in low and high wealth areas, within the same population group, reduces as we move the focus from "low-wealth" population to "high-wealth" population. Consistently with the results discussed in the main text, for all three groups of individuals based on their residential area, those working in high-wealth administrative units always show a higher degree of change in the fraction of individuals not-commuting to work.

“Low-wealth” population

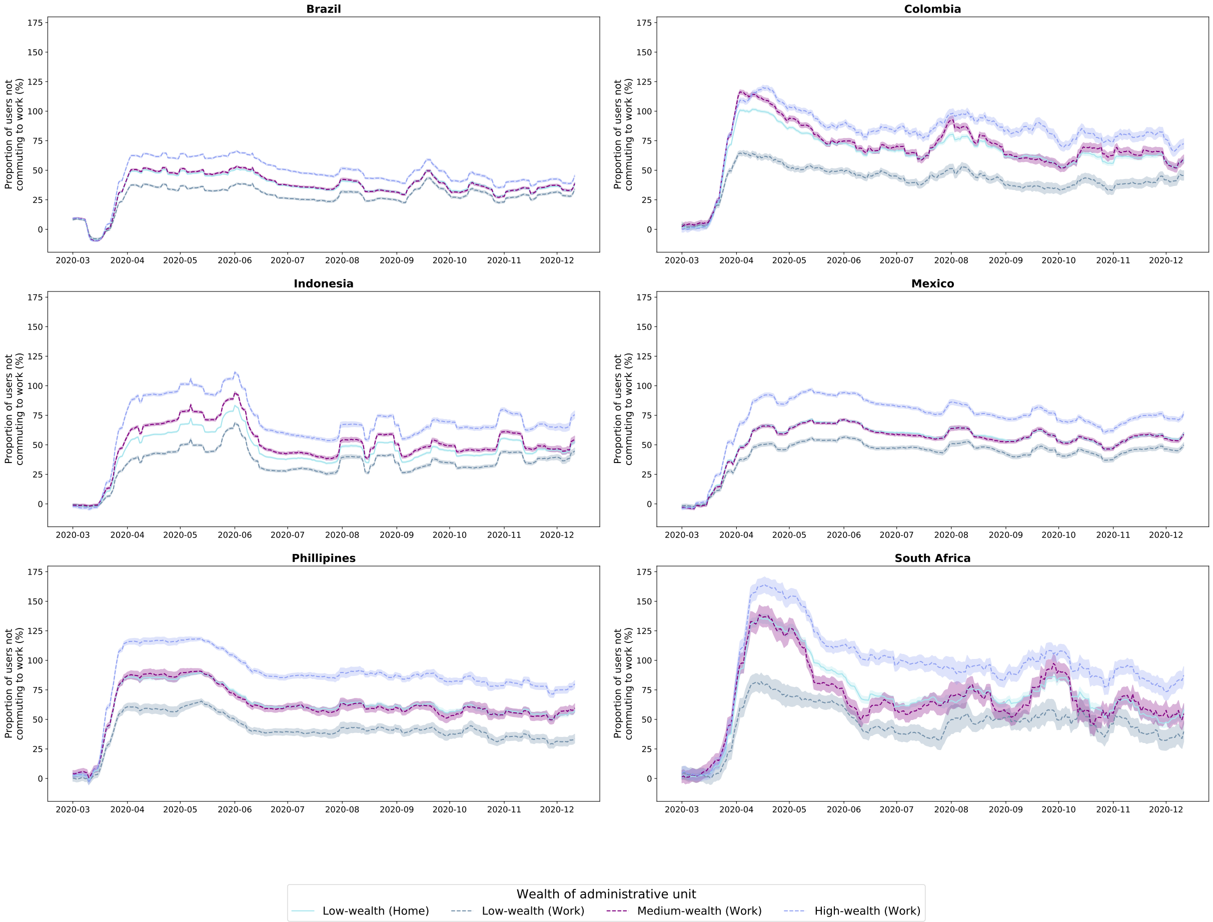

Figure SI 15. Change in the fraction of low-wealth users not commuting based on the wealth of their workplace area.

“Medium-wealth” population

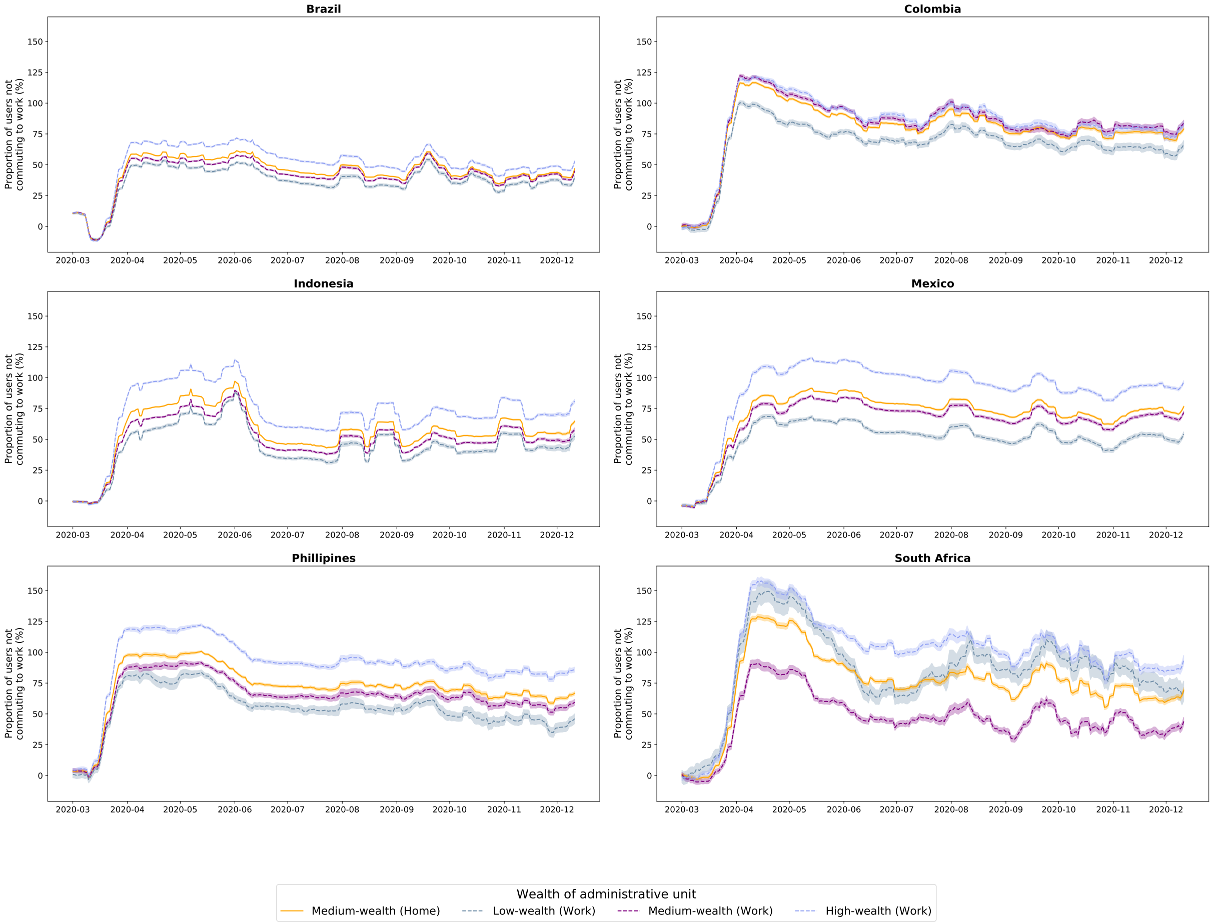

Figure SI 16. Change in the fraction of medium-wealth users not commuting based on the wealth of their workplace area.

“High-wealth” population

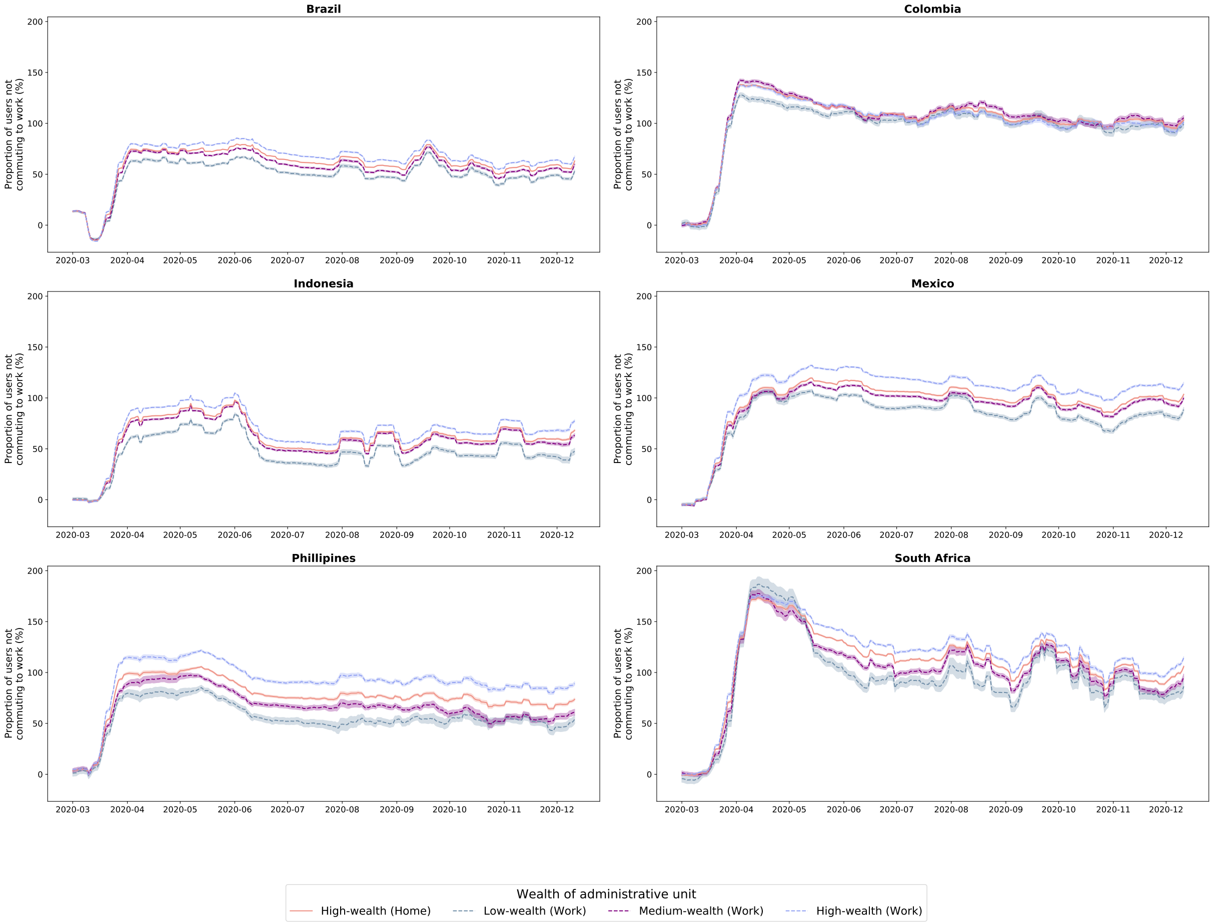

Figure SI 17. Change in the fraction of high-wealth users not commuting based on the wealth of their workplace area.

SI 11. GROUP SPECIFIC AND ASPECIFIC MOBILITY BASELINES AND MOBILITY BEHAVIOR CHANGES DURING 2020

In the main text of this work, we discussed how individuals' behavior abruptly changed across multiple countries at the time when the COVID-19 pandemic was declared. The analysis focused on group-specific changes with respect to group-specific baseline behavior. In this section, we provide additional evidence in support of the findings discussed in the main manuscript.

A. Mobility indicators over time using a common baseline.

In this section, we focus on self-isolation patterns (in terms of the relative fraction of individuals not leaving home for an entire day) and commuting patterns (in terms of the relative change in the fraction of individuals visiting their work location on a specific day). For both mobility indicators, we explore wealth-group behavior over the entire observation period. Figures SI 18 - SI 19 report the changes in self-isolation and commuting patterns over the entire period showing a systematic reduction in mobility after the pandemic declaration. Furthermore, Fig. SI 18 and Fig. SI 19 highlight how group-specific mobility behavior, when compared with the population behavior, consistently shows smaller changes for individuals living in low-wealth neighborhoods. The two figures also reveal how mobility differences between wealth groups were present also before the pandemic. However, these inherent social mobility differences appear to be overruled once the pandemic kicked in.

B. Self-isolating, non-commuters, and relocation to rural areas

As in the main manuscript we provided the reader with relative change information about mobility behavior indicators, in this section we provide average behavior during the baseline period as well as during the pandemic. Absolute average numbers nicely complement relative changes by providing a way to quantify the raw effect of the pandemic on mobility patterns. In Table I, we report in each column the information for a specific country. Rows are divided into blocks each of which focuses on a specific mobility indicator: the number of individuals isolating at home, the number of individuals not visiting their work location, and the number of individuals who changed home location with respect to the home location detected during the baseline period. The numbers are the average over all days within the baseline and the pandemic period of the number of individuals. As relocation refers to changes in home location with respect to the home location during the baseline period, no relocation can occur by definition during the baseline period. Similarly, in Table II we report the percentages of users isolating at home and the percentage of users commuting to

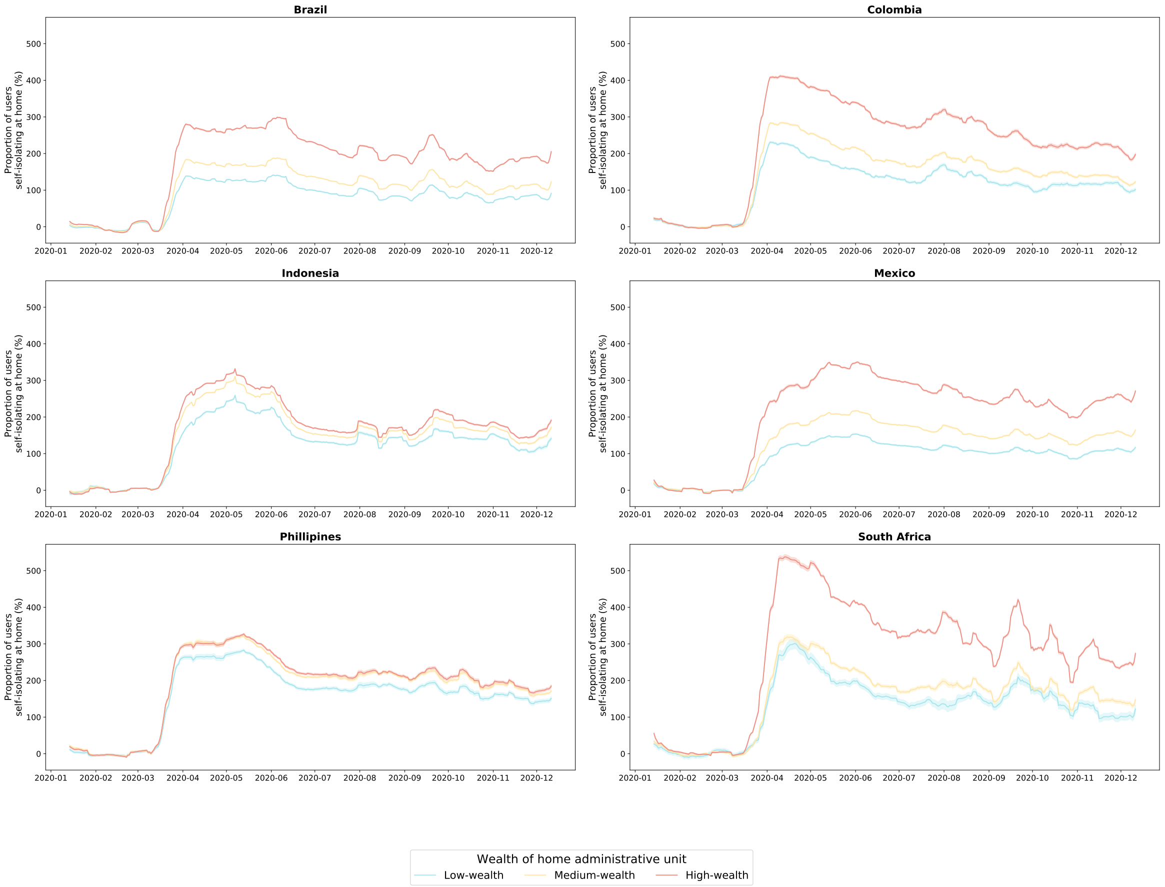

Figure SI 18. *Self-isolation group-specific mobility behavior changes over the entire observation period.* Relative change is computed in terms of the mobility behavior of each wealth group separately during the pre-pandemic period.

work during the baseline period, to act as a reference for better quantifying the overall behavioral change size when looking at relative change measures. Percentages are reported for each country and each wealth group.

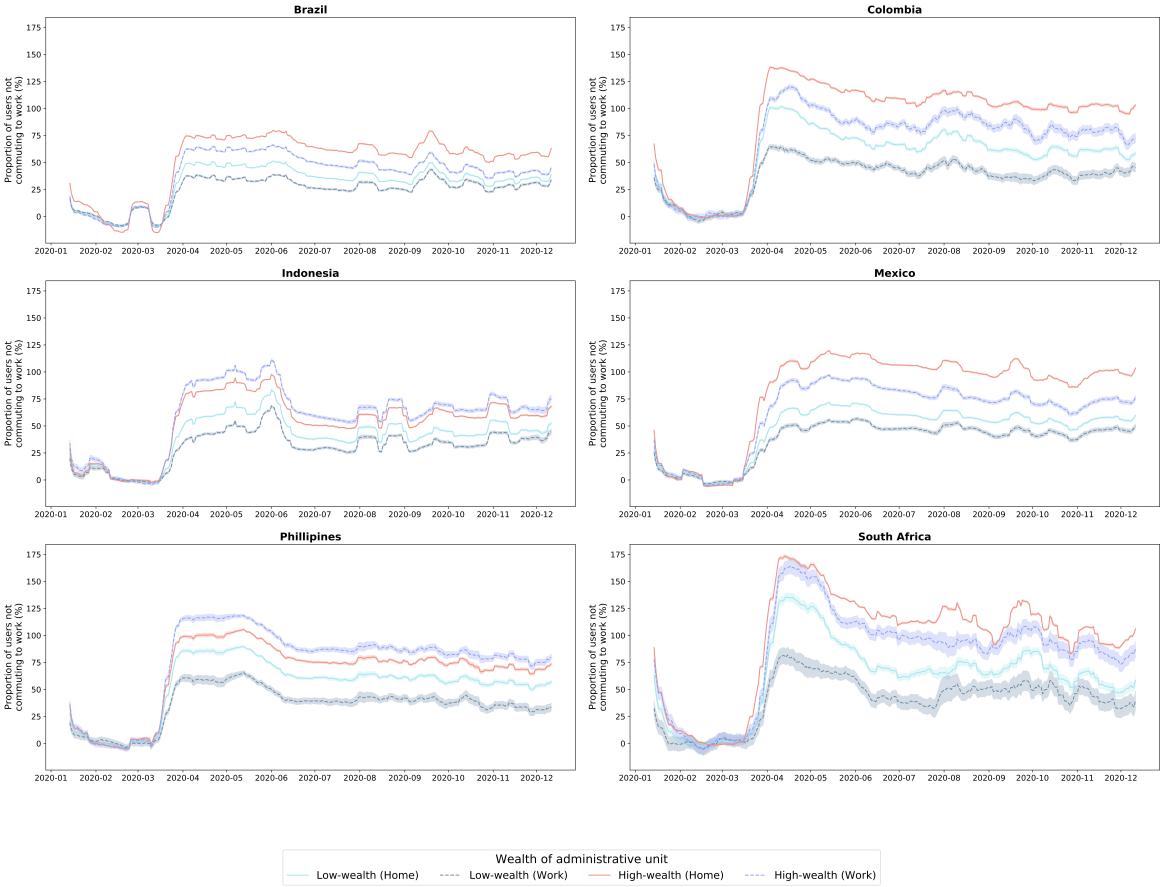

Figure SI 19. *Commuting to work group-specific mobility behavior changes over the entire observation period.* Relative change is computed in terms of the mobility behavior of each wealth group separately during the pre-pandemic period.

C. Population-representative mobility as a null model for wealth-specific mobility behavior

In the main manuscript we reported the change in the fraction of individuals who are not commuting from home to work focusing on the behavior of individuals living in "low-wealth" neighborhoods. Here we assume as a null model the scenario in which each individual has its corresponding wealth label reshuffled. The random assignment of labels and subsequent aggregation of mobility patterns results in homogenizing the mobility behavior of wealth groups to the average population-representative mobility behavior. This section show that the mobility behavior of the medium-wealth group is closer to the behavior of a representative population disregarding wealth effects. The three figures (Fig. SI 22, Fig SI 23, and Fig. SI 24) respectively show the results of i) the change in the share of users self-isolating at home, ii) the net share of urban users relocating to rural areas, and iii) the change in the fraction of users not commuting by socioeconomic group. In all figures we report in black the population-representative null comparison (mean).

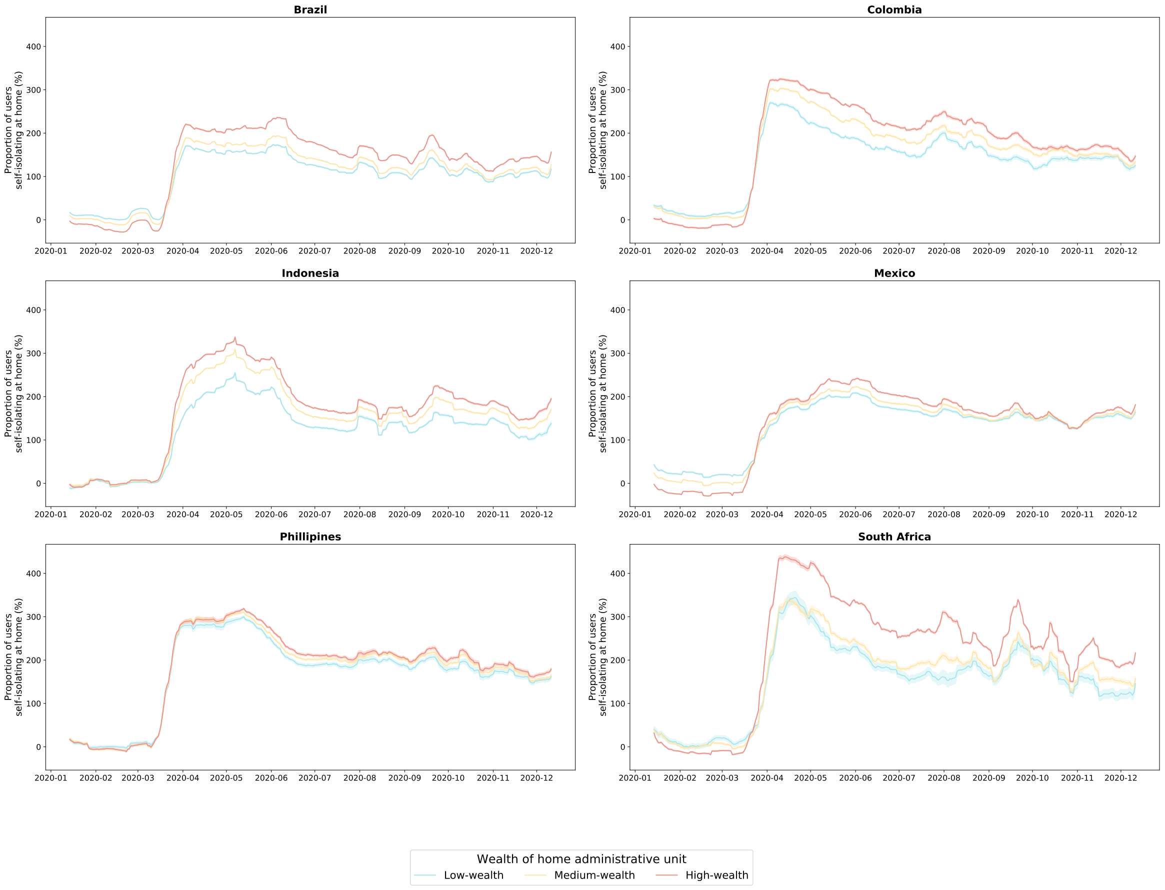

Figure SI 20. *Self-isolation group-specific mobility behavior changes over the entire observation period.* Relative change is computed in terms of the mobility behavior of each country's entire population (within our dataset) during the pre-pandemic period.

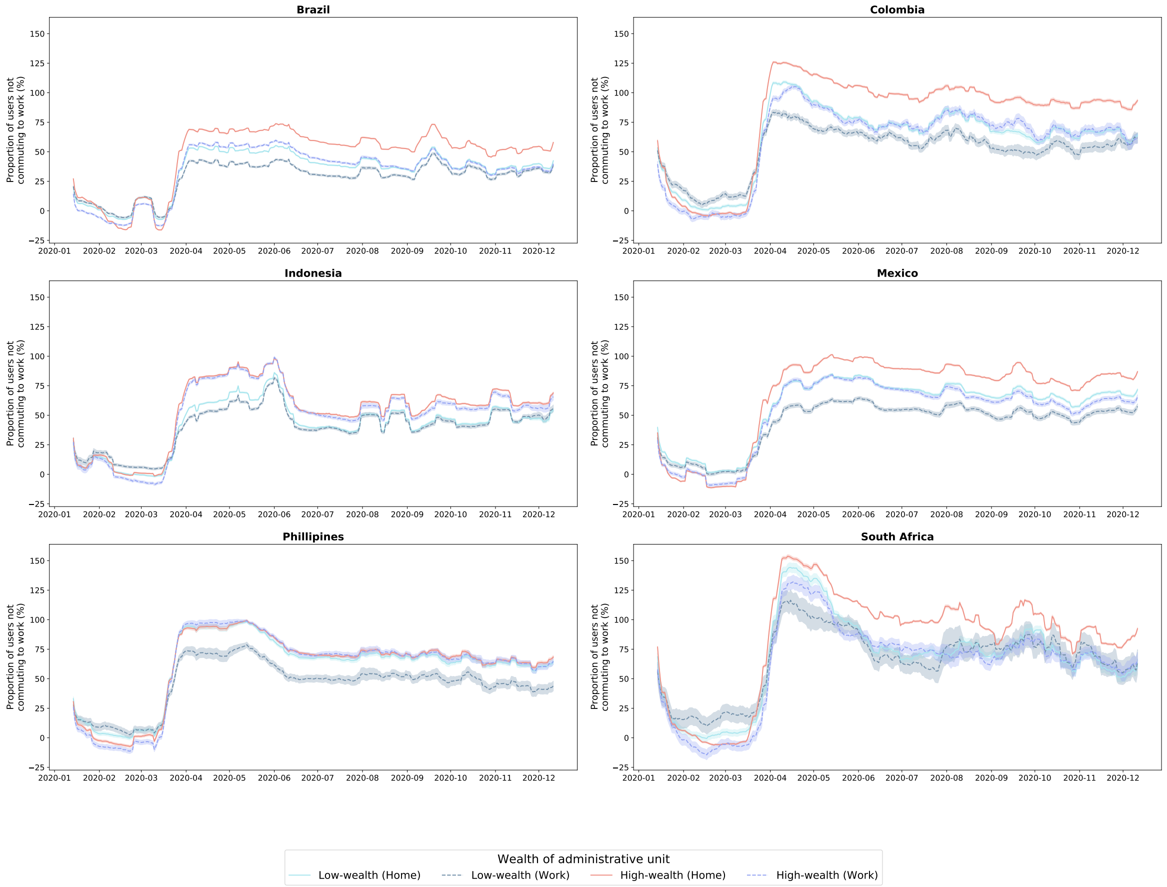

Figure SI 21. *Commuting to work group-specific mobility behavior changes over the entire observation period*. Relative change is computed in terms of the mobility behavior of each country's entire population (within our dataset) during the pre-pandemic period.

Table I. Average Number of Individuals Isolating and Not Commuting During Baseline and Pandemic Periods by Wealth Group and Country. Relocation data only refers to the pandemic period as migration is defined based on changes in the home location detected during the baseline period.

Group	Period	BR	CO	ID	MX	PH	ZA
**Isolating**							
High	Pre	6225.08 (±568.06)	663.00 (±63.87)	4829.57 (±477.66)	2471.07 (±260.77)	753.10 (±86.70)	1742.79 (±197.74)
	Post	10569.86 (±302.16)	1183.98 (±34.71)	8511.82 (±273.90)	4893.58 (±153.90)	1211.80 (±44.97)	3461.80 (±106.44)
Medium	Pre	7416.62 (±654.25)	799.30 (±72.36)	5973.28 (±605.18)	2838.13 (±277.96)	735.50 (±84.01)	469.06 (±43.63)
	Post	9301.21 (±259.03)	1071.42 (±31.72)	10151.27 (±336.53)	4213.73 (±128.28)	1170.04 (±43.06)	682.55 (±18.30)
Low	Pre	5418.00 (±465.06)	446.24 (±40.12)	1942.71 (±188.78)	1687.76 (±156.55)	371.44 (±40.40)	125.39 (±11.83)
	Post	5872.44 (±162.37)	519.44 (±15.64)	3117.24 (±101.67)	2067.68 (±61.72)	557.00 (±19.97)	177.24 (±4.74)
**Not Commuting**							
High	Pre	17804.54 (±1623.74)	1736.68 (±163.22)	13697.06 (±1327.18)	7206.86 (±744.51)	1764.29 (±197.04)	4910.81 (±499.30)
	Post	15858.54 (±442.18)	1642.64 (±46.18)	13274.47 (±409.41)	7588.30 (±229.37)	1578.57 (±57.13)	5153.15 (±153.42)
Medium	Pre	17966.88 (±1579.53)	1725.46 (±154.93)	16708.04 (±1654.76)	6635.67 (±647.95)	1737.92 (±191.27)	1184.33 (±103.36)
	Post	14403.40 (±393.44)	1491.55 (±42.35)	16170.35 (±515.90)	6429.34 (±190.17)	1546.93 (±55.62)	1098.98 (±29.08)
Low	Pre	12092.89 (±1028.43)	921.27 (±81.04)	5674.26 (±541.48)	3495.27 (±324.58)	866.49 (±91.50)	300.81 (±26.37)
	Post	9228.75 (±250.71)	743.66 (±21.60)	5380.85 (±170.61)	3139.18 (±91.68)	757.79 (±26.54)	286.33 (±7.63)
**Relocating to Rural Areas**							
High	Pre	NA	NA	NA	NA	NA	NA
	Post	1605.10 (±41.63)	247.68 (±6.00)	1001.06 (±19.62)	848.92 (±20.14)	145.07 (±2.79)	478.47 (±20.89)
Medium	Pre	NA	NA	NA	NA	NA	NA
	Post	661.64 (±15.92)	120.39 (±2.99)	718.10 (±14.86)	620.37 (±12.29)	133.78 (±2.46)	53.04 (±3.24)
Low	Pre	NA	NA	NA	NA	NA	NA
	Post	292.30 (±8.21)	45.22 (±1.30)	174.24 (±3.92)	232.69 (±4.91)	44.68 (±0.84)	16.24 (±0.75)

Table II. Average percentage of Individuals Isolating and Not Commuting During Baseline Periods by Wealth Group and Country.

		BR	CO	ID	MX	PH	ZA
	**wealth group**						
**Daily share of people isolating**	High	32.55 %	39.71 %	32.12 %	38.8 %	39.83 %	30.86 %
	Low	21.84 %	24.72 %	18.42 %	25.21 %	25.95 %	17.46 %
	Medium	30.57 %	36.52 %	27.56 %	36.45 %	38.48 %	24.49 %
**Daily share of people commuting**	High	30.35 %	35.43 %	33.88 %	36.65 %	32.73 %	37.65 %
	Low	31.07 %	42.81 %	37.68 %	41.97 %	36.55 %	47.66 %
	Medium	22.25 %	29.16 %	25.95 %	29.71 %	25.21 %	38.97 %

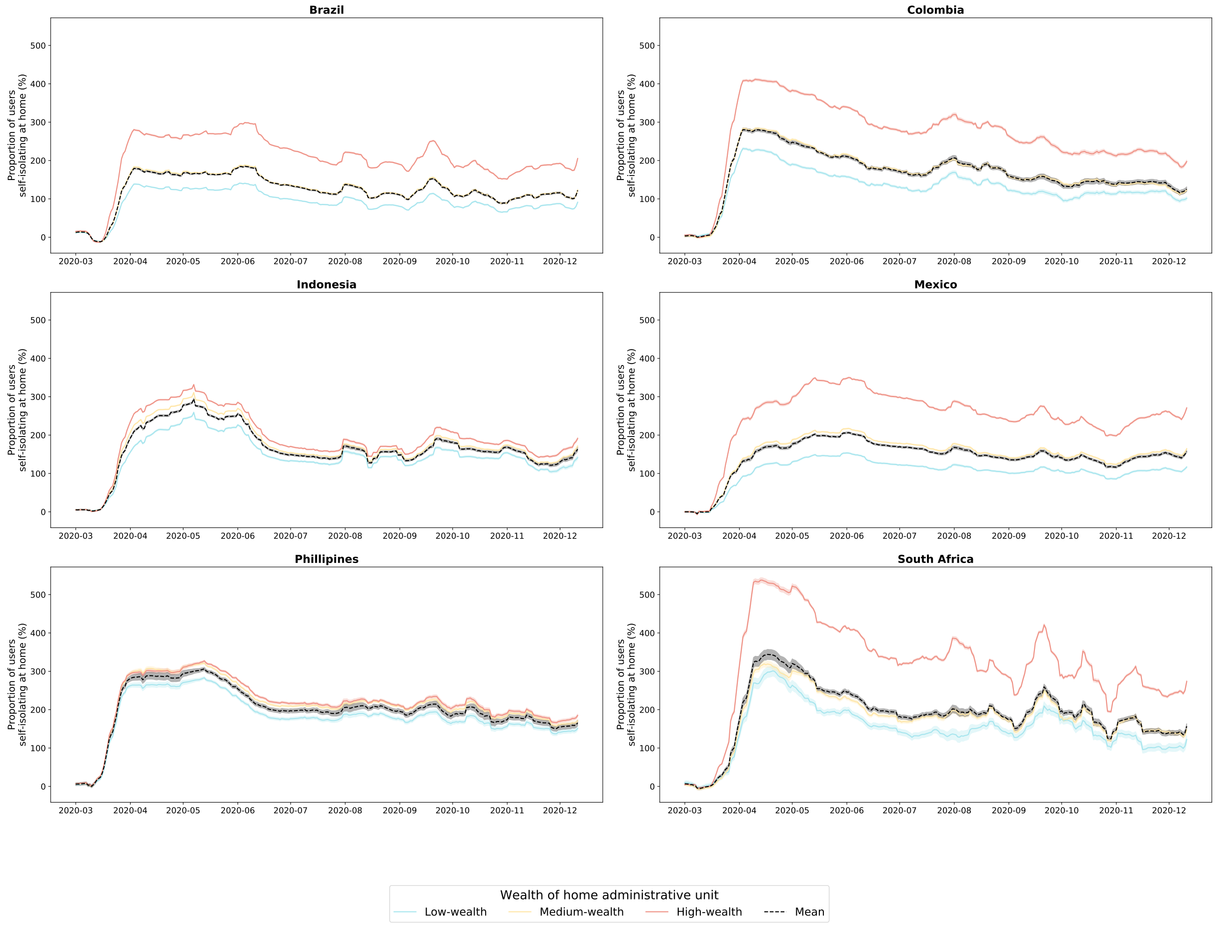

Figure SI 22. Change in the share of users self-isolating at home by socioeconomic group with a population-representative null comparison (mean).

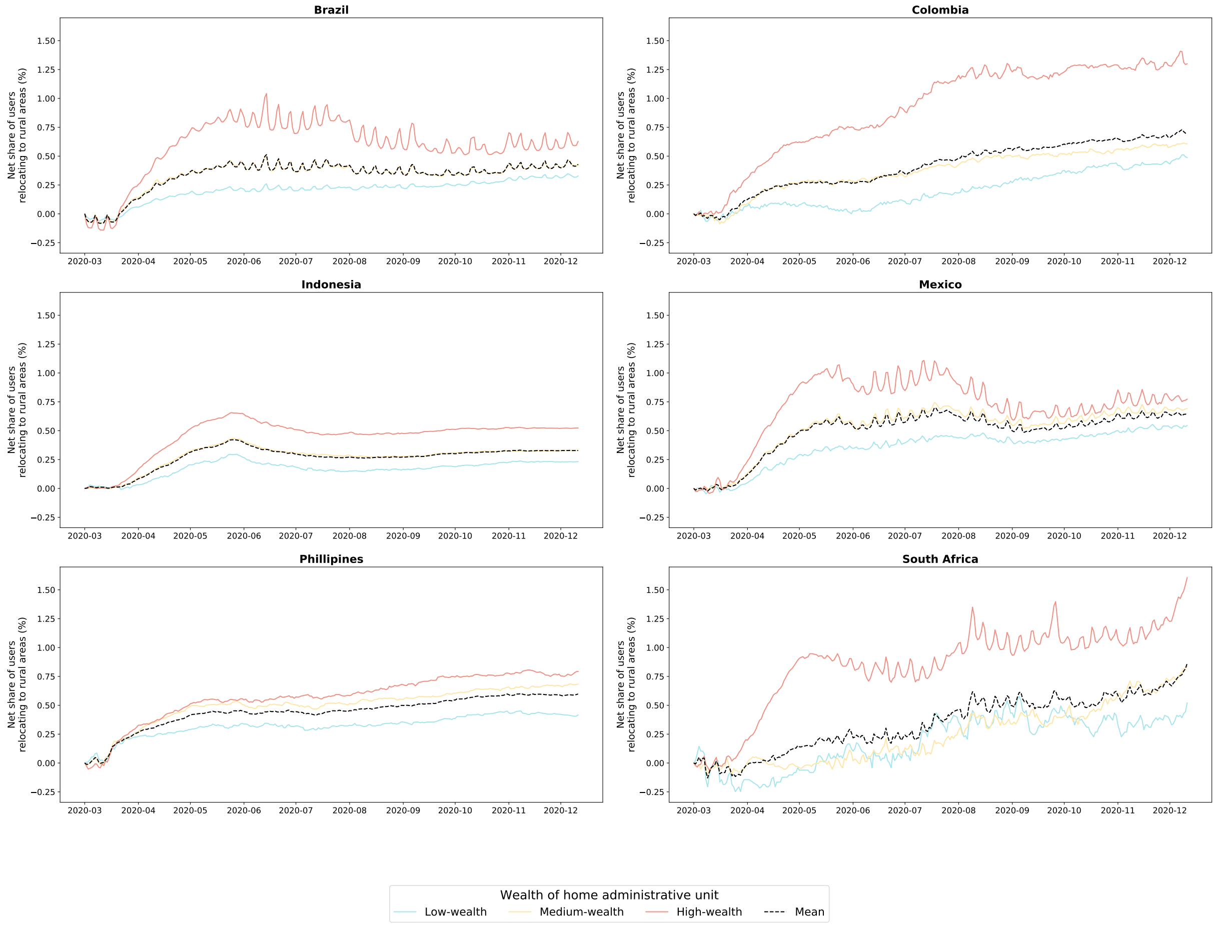

Figure SI 23. Net share of urban users relocating to rural areas by socioeconomic group with a population-representative null comparison (mean).

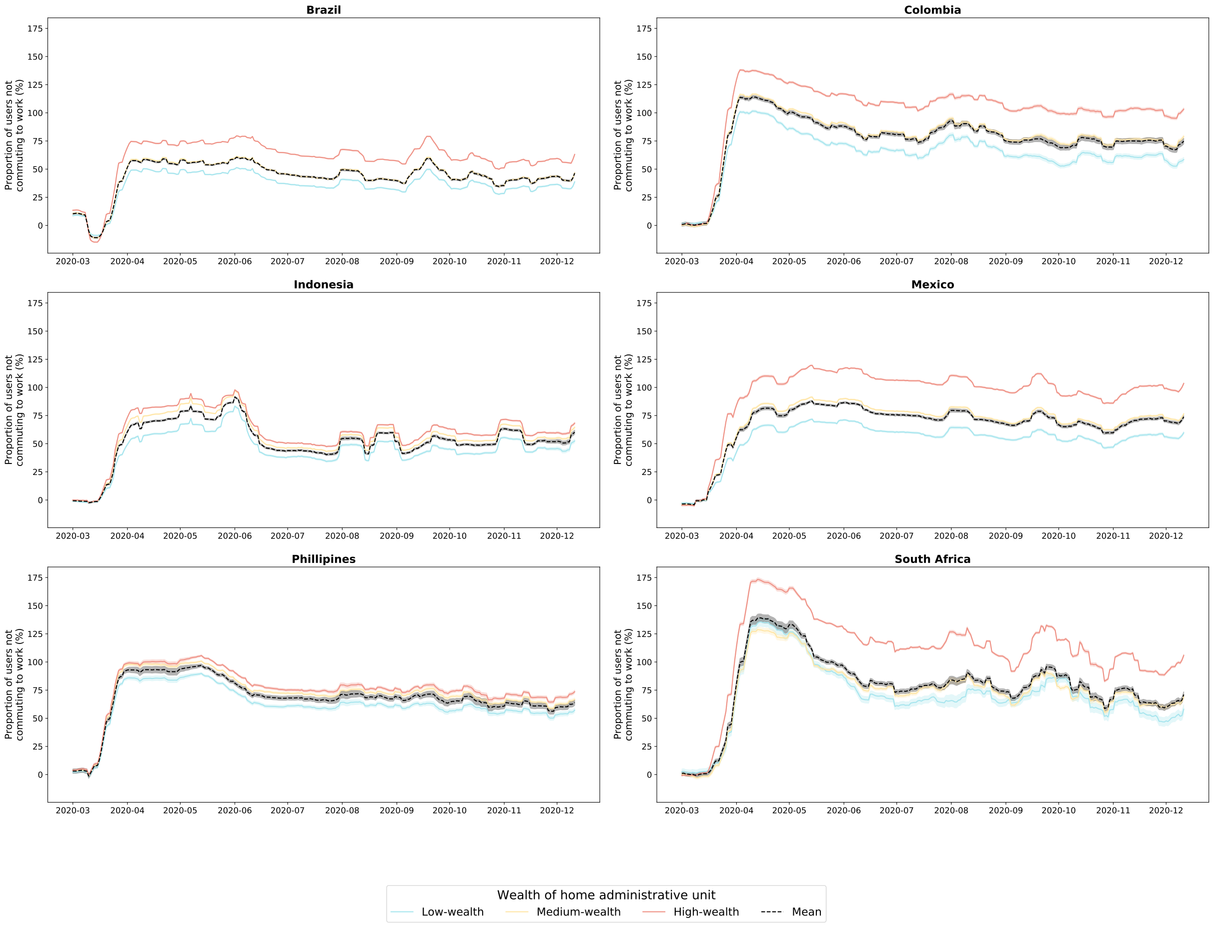

Figure SI 24. Change in the fraction of users not commuting by socioeconomic group with a population-representative null comparison (mean)

SI 12. MOBILITY BEHAVIOR AND POLICY INTERVENTIONS

A. Hierarchical model components selection

To provide policy-specific associations we first divide policies into three different categories as labeled by Hale et al. [8]: containment, economic, and health-related policies. Containment policies are those focused on reducing physical contact among citizens: school, workplace, public transport closures, stay-at-home orders, and internal movement restrictions. Economic policies are those providing economic support to citizens either via debt relief or income support. Health-related measures are those aimed at informing about safe behavior (information campaigns), tracing and testing, and other preventive behavior, such as facial covering and protection of elderly people. Epidemiological quantities such as local case incidence and local death incidence are included as covariates. The model is configured as a panel regression model, where group-and-country-specific mobility indicators are associated with local incidence and policy-categories indices. Following the same notation as in the main manuscript:

$$
\begin{aligned}
mb_{ic}(t) = & a_i * incidence_g(t) && (1)\\
& + b_i * incidence_c(t) && (2)\\
& + c_{i,contain} * C_{c,contain}(t) && (3)\\
& + c_{i,economic} * C_{c,economic}(t) && (4)\\
& + c_{i,health} * C_{c,health}(t); && (5)
\end{aligned}
$$

We run a generalized least-square regression to estimate model parameters and we compute the BIC as a metric for model performance comparison. We compare this model with simpler models, involving fewer covariates to only keep significant ones. In particular, we compare all possible combinations of the five covariates presented in Eq.1-4.

Figure SI 25 reports BIC values for all model combinations. All models that are included do not show significant multicollinearity. Multicollinearity is tested by means of the Variance Inflation Factor (VIF) [10], and is required to be below 4 for each component in the model. In this perspective, focusing on the period starting from April 2020 helps reduce multicollinearity between different policy indices, as it discards the simultaneous early-policies enactment. This, in turn, makes it possible to more precisely estimate regression coefficients.

Further robustness testing is performed by regressing the models on single country data (see Fig. SI 26-SI 31). Consistent results are found, showing that both global incidence of cases, local incidence of cases,

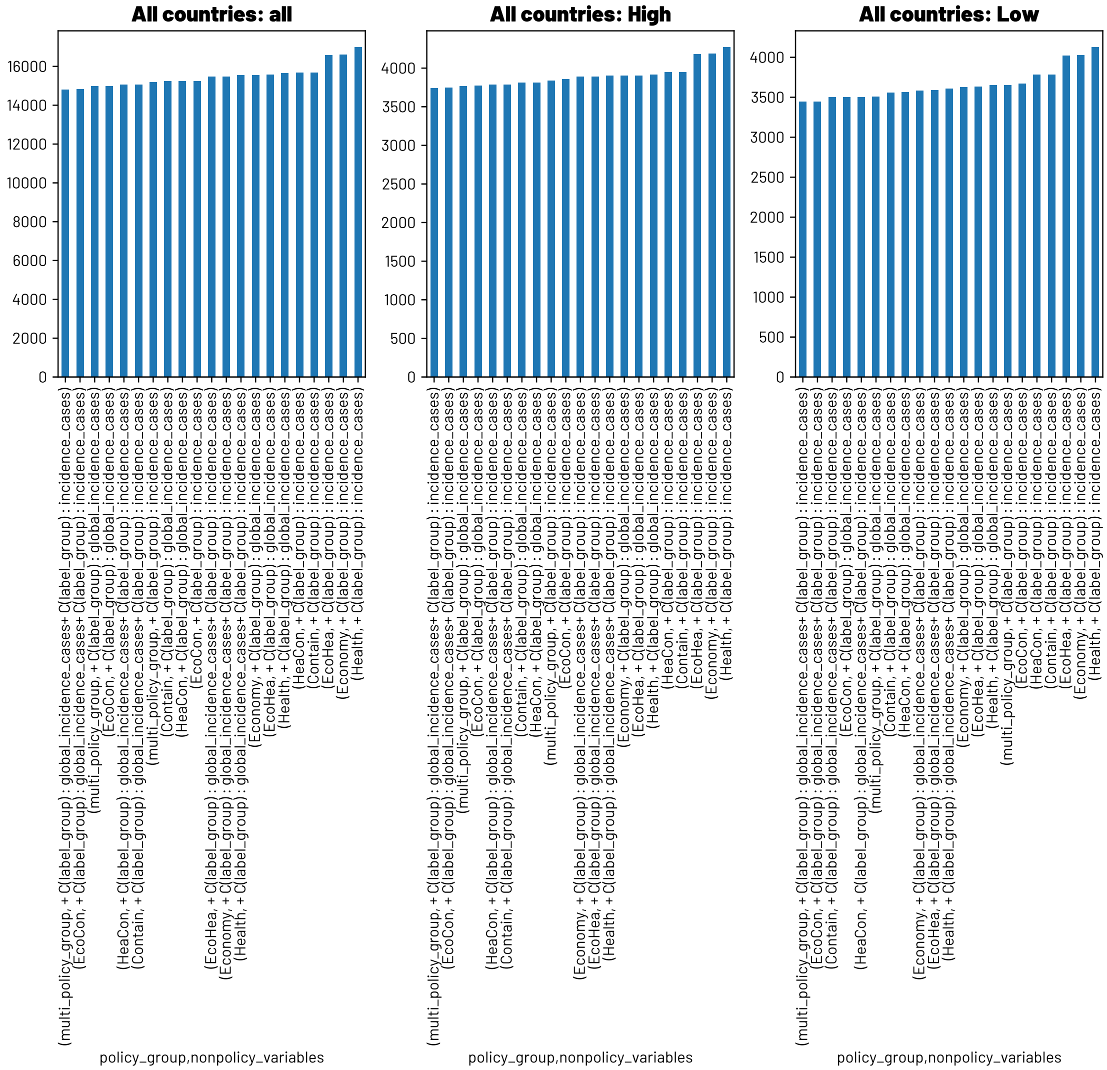

Figure SI 25. *BIC values for all model combinations that do not show any significant multicollinearity.* Each panel reports BIC values when modeling behaviour of "all" wealth groups (left), "high-wealth" group only (center), and "low-wealth" group only (right).

and Containment policies play a crucial role in modeling mobility behavior.

To more precisely understand single policies association with mobility behavior we would need to disaggregate policy indices into single policy types. However, due to the limited amount of policy activation and loosening over 2020, strong multicollinearity arises by including all single-policy indices without any further selection. To overcome this issue, we focus our attention on Containment policies which are found to be hierarchically the most important policy group in modeling mobility behaviour.

The containment index is thus disaggregated into 5 different single-policy indices $(C_1(t), C_2(t), \ldots, C_5(t))$ which include: school closure, workplace closure, public transport closure, stay-at-home requirements, and internal movement restriction policies.

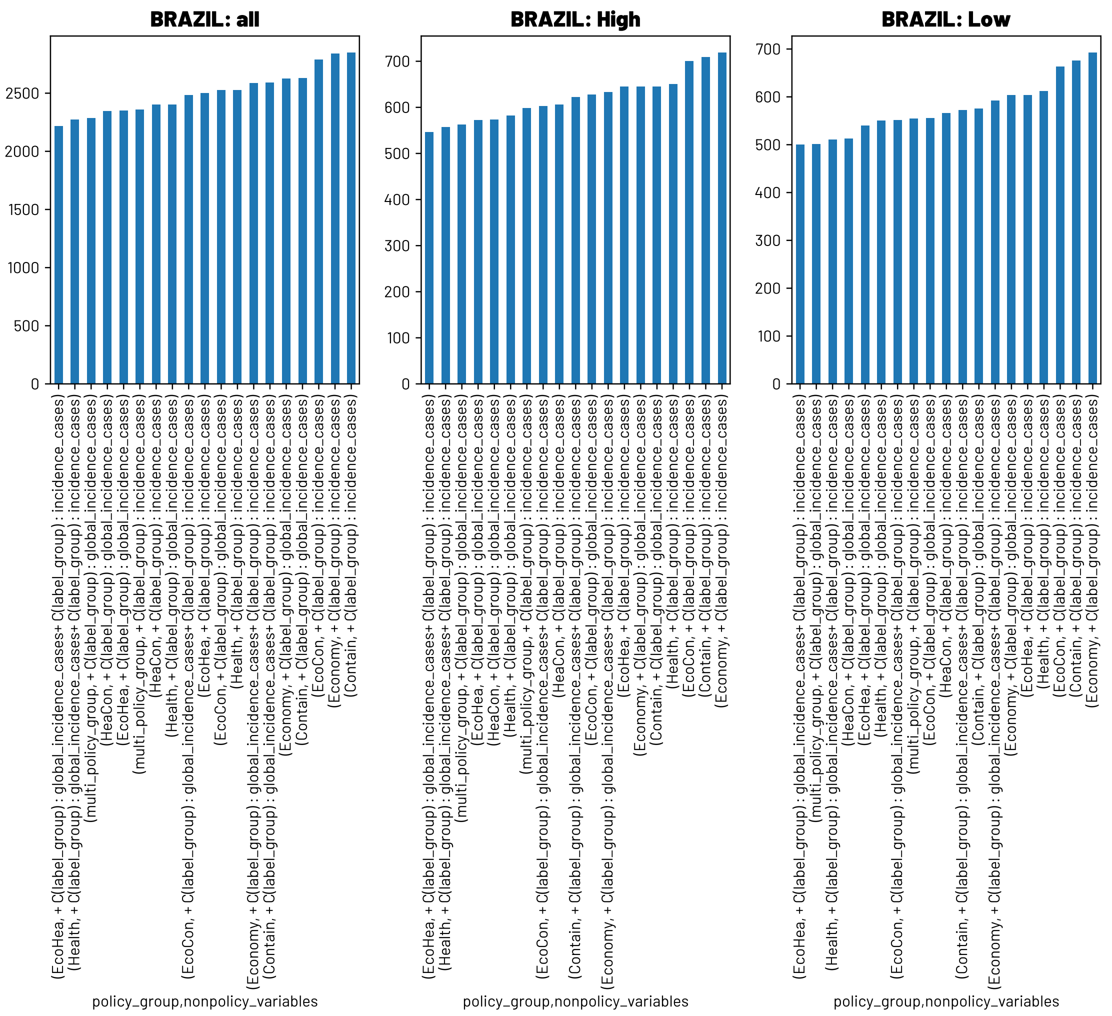

Figure SI 26. *BIC values for all model combinations that do not show any significant multicollinearity*. Regression is performed only on Brazil's mobility and epidemiological data.

B. Robustness tests for single-policy indices' model

In this section, we perform multiple modeling robustness tests to check the results' reliability. In particular, we are interested in confirming the robustness of the different association levels between low-wealth individuals working in high-wealth neighborhoods and low-wealth individuals working in low-wealth neighborhoods. Results reported in the main manuscript show that all considered policies, except for closures of public transport, do not have a disproportionate association with a wealth-specific behavioral response when looking at commuting behavior.

To this end, we structure this section as follows. First, we test that changing the time window over which the regression is performed does not hamper our results, provided that a large enough time span is included to have a minimum amount of policy index changes to be analyzed. Second, we test our model

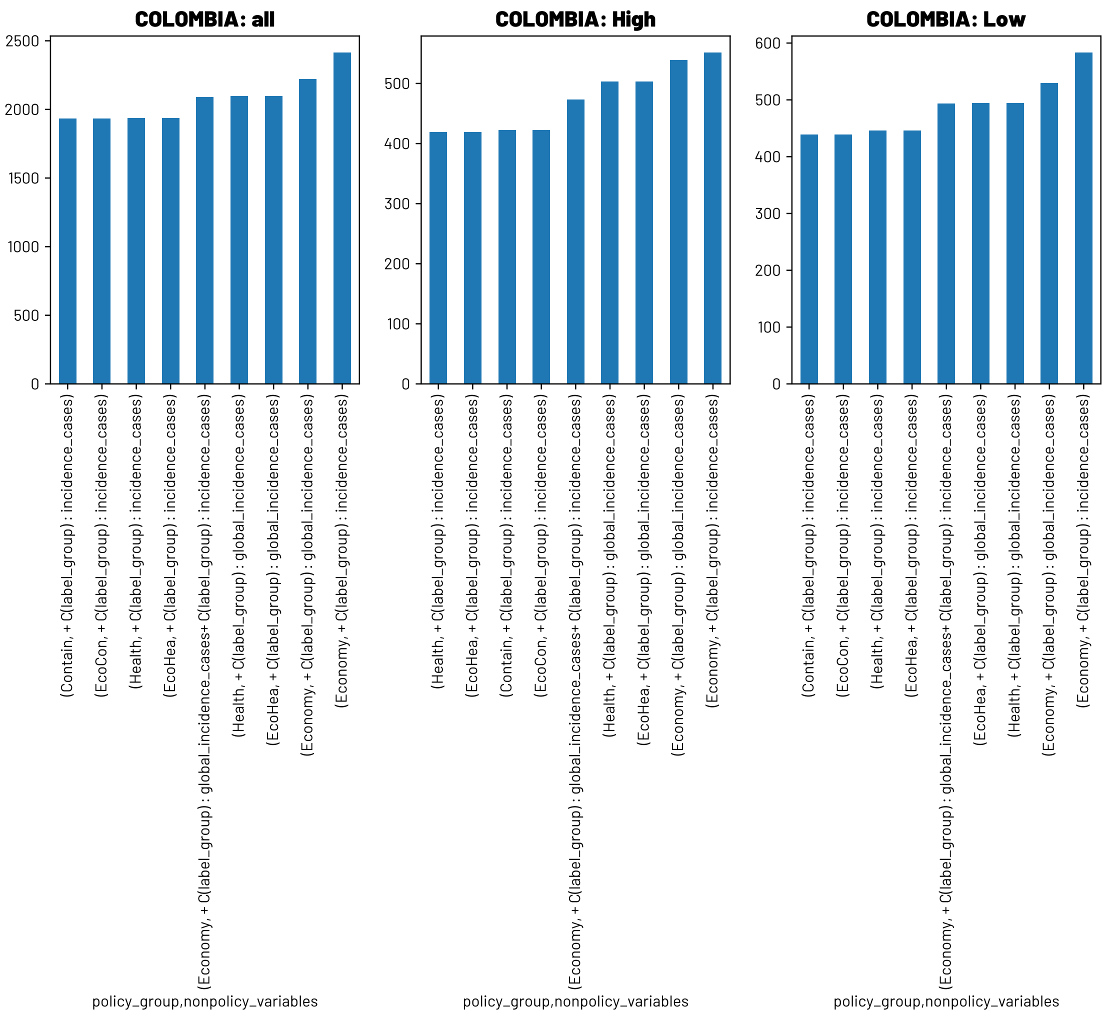

Figure SI 27. *BIC values for all model combinations that do not show any significant multicollinearity*. Regression is performed only on Colombia's mobility and epidemiological data.

including also other variables from different policy group types. Namely, we report parameter estimates for the single-policy regression including Containment and Economic single-policy indices, as it is the highest-ranked model with more than one policy group. Third, we check results robustness when only one epidemiological variable is used in the model.

The results presented in the main paper are recovered in all robustness tests for which enough statistics are available. Countries with few changes in specific single-policy indices do not always provide significant results, but average results values show consistency with the results reported in the main manuscript.

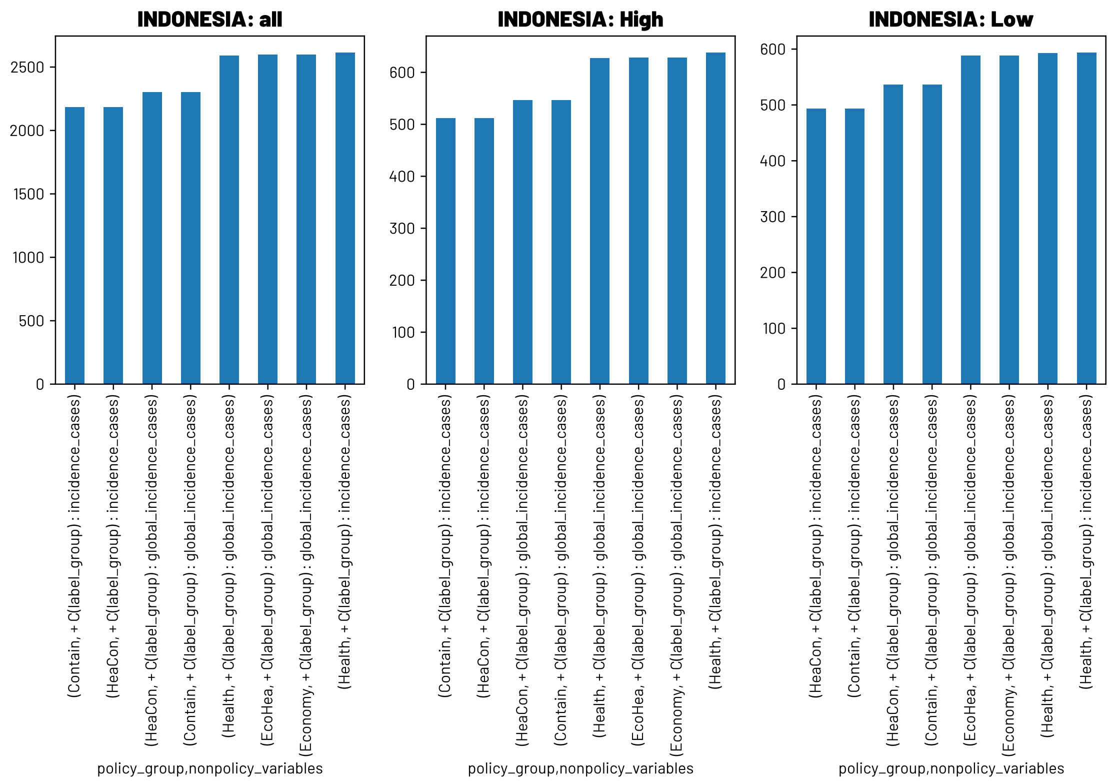

Figure SI 28. *BIC values for all model combinations that do not show any significant multicollinearity*. Regression is performed only on Indonesia's mobility and epidemiological data.

1. Modeling on different periods

We focus our attention on two different periods: the first includes only early pandemic period, spanning from February 11, 2020, to May 10, 2020; the second spanning from February 11, 2020, to the end of the year.

Results show that including the early period of the pandemic in the regression we recover the results discussed in the main manuscript, where parameters are estimated starting from April 11, 2020, until the end of 2020. In particular, the entire pandemic period during 2020 returns significantly different coefficient values for the high-wealth and the low-wealth groups (see Fig. SI 32-left). We stress that the choice of the time window negatively impacts the reliability of the model in the case of the early months of the pandemic, Fig. SI 32-right. High levels of multicollinearity are found if only those months are included in the regression. Nevertheless, we always find significant differences in transport closures' values between high-wealth and low-wealth groups, with high-wealth having greater parameter values.

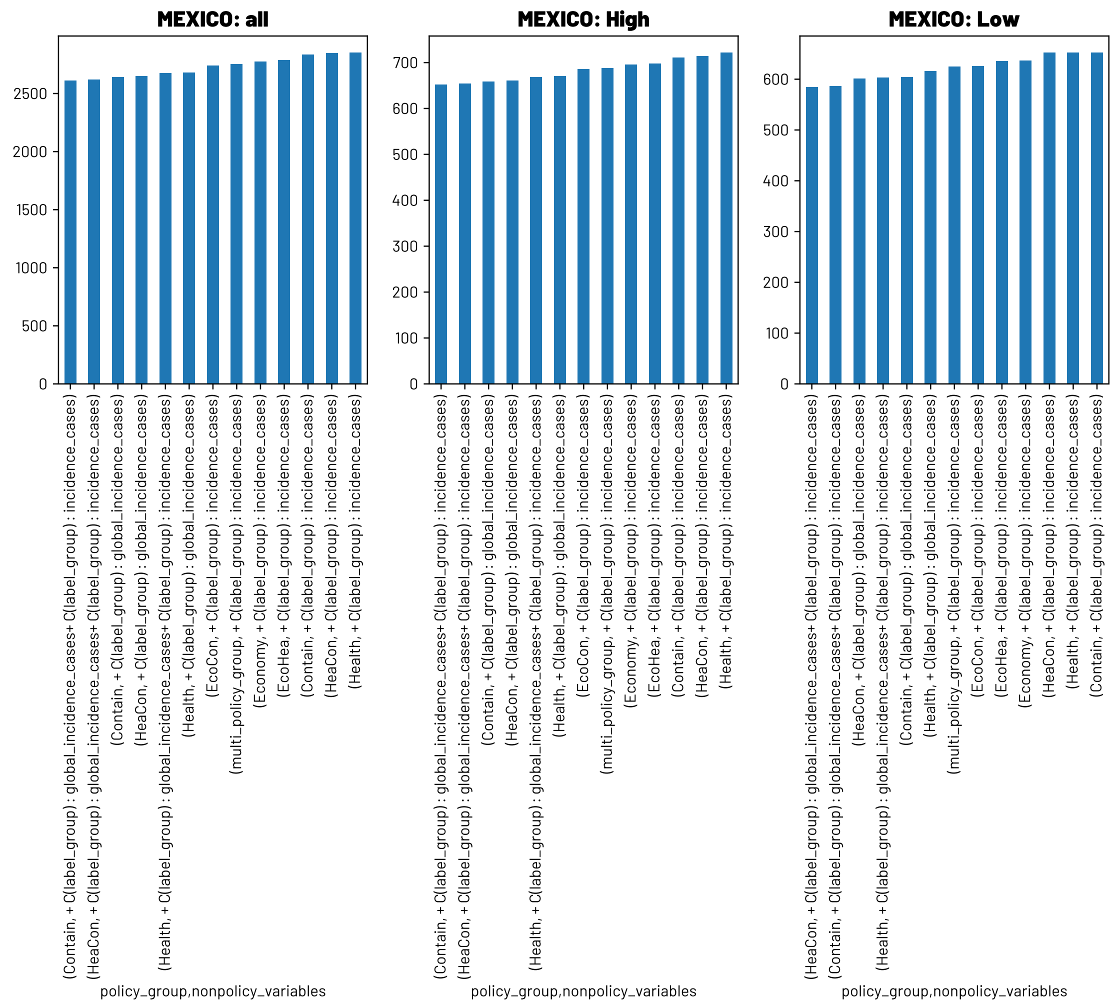

Figure SI 29. *BIC values for all model combinations that do not show any significant multicollinearity*. Regression is performed only on Mexico's mobility and epidemiological data.

2. *Cross-validation on different country sets*

To further test the robustness of our results, we perform our regression analysis on different subsets of countries, removing one country at a time.

Results show that, while significant differences are not always recovered, the difference in the estimate of the parameters is always greater for low-wealth individuals working in high-wealth neighborhoods. We stress that also in this case, limiting the regression to a smaller set of countries results in higher levels of multicollinearity. Nevertheless, all covariates in the models show VIF scores smaller than 4.

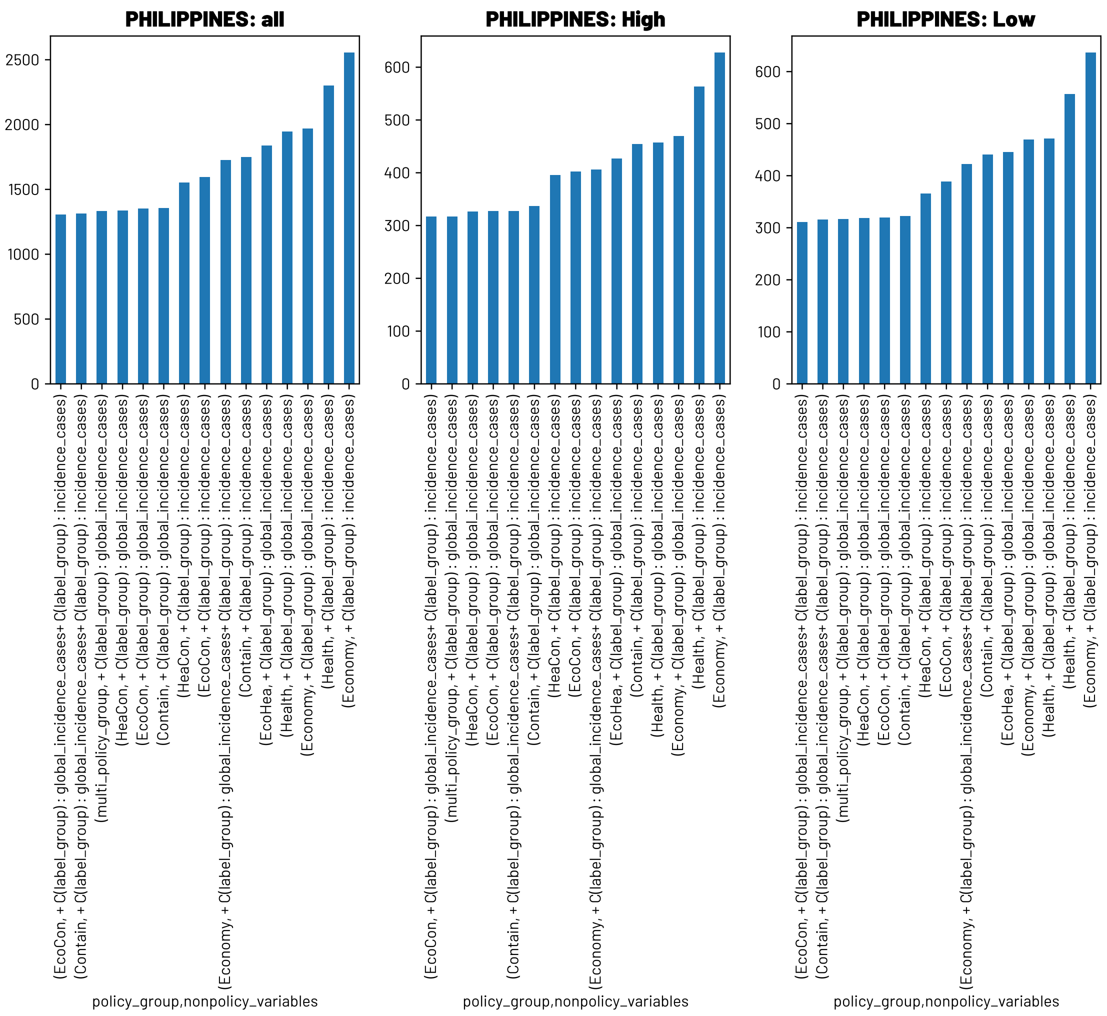

Figure SI 30. *BIC values for all model combinations that do not show any significant multicollinearity*. Regression is performed only on Philippines mobility and epidemiological data.

3. *Modeling with additional policy indices*

We also test our results against the addition of more policy indices. In particular, following the procedure in Sec. SI 12 A, we select the second-best model (in terms of BIC values) including two groups of policy types: Containment and Economic policies. These two policy groups are divided and each single-policy index is included in the model. In particular, the economy single-policy indices that are added are i) income support policies, and ii) debt or contract relief policies (see [8, 14] for more details on the definition of those groups). Figure SI 34 shows that both indices are negatively associated with the suspension of commuting patterns. However, no significant difference between wealth groups is found. In contrast, public transport policies are found to be significantly associated with different parameter values depending on the wealth groups, with low-wealth individuals working in high-wealth neighborhoods being more strongly associated

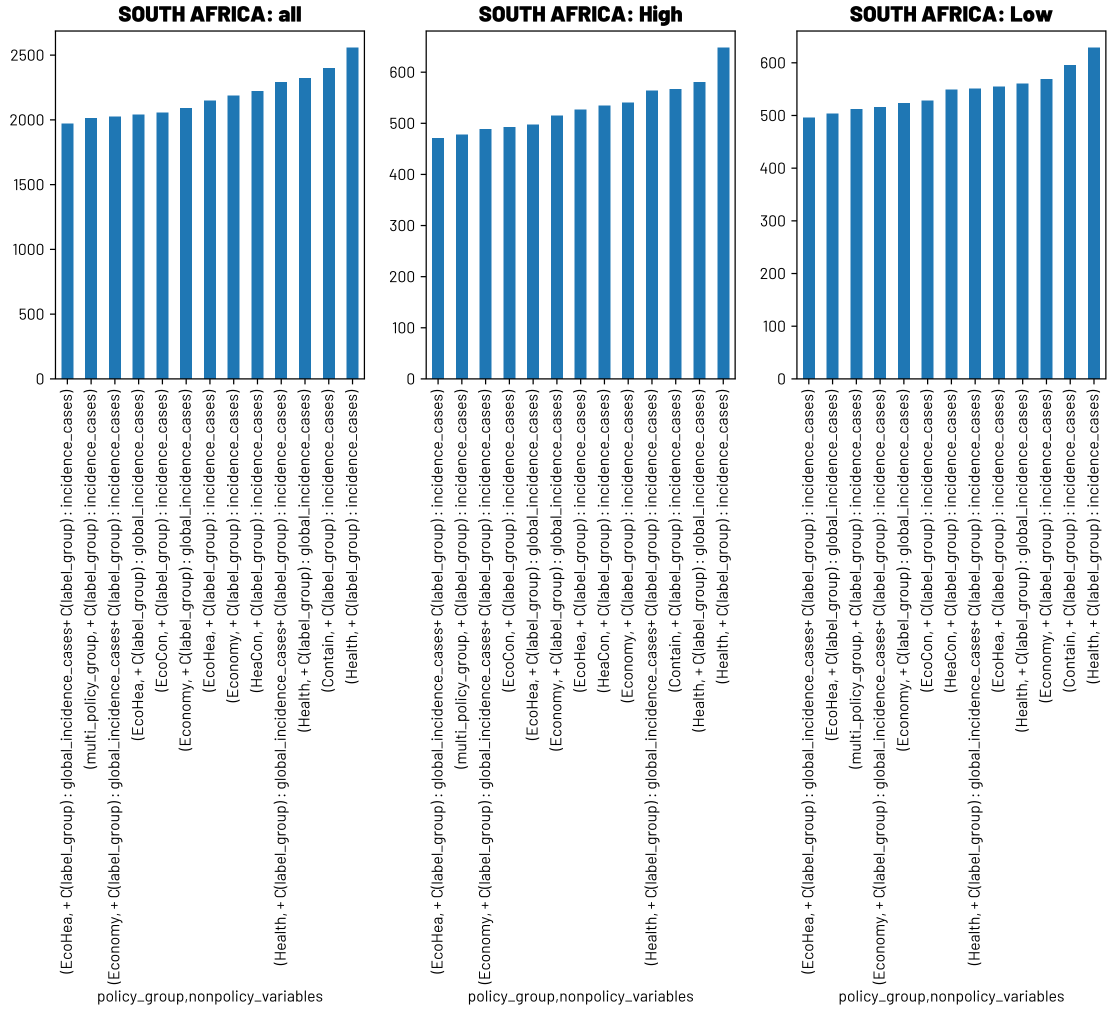

Figure SI 31. *BIC values for all model combinations that do not show any significant multicollinearity*. Regression is performed only on South African mobility and epidemiological data.

than low-wealth individuals working in low-wealth neighborhoods.

4. *Incidence of cases: local and global effects on model*

To further test the robustness of our results, we perform our regression analysis with two different models: the first one only includes the global incidence of cases, while the second one only includes the local (country-specific) incidence of cases. In the model discussed in the main manuscript, both epidemiological indicators were included. Results hold in both cases, as shown in Fig. SI 35-left (for global incidence of cases only) and in Fig. SI 35-right (for country specific incidence of cases only).

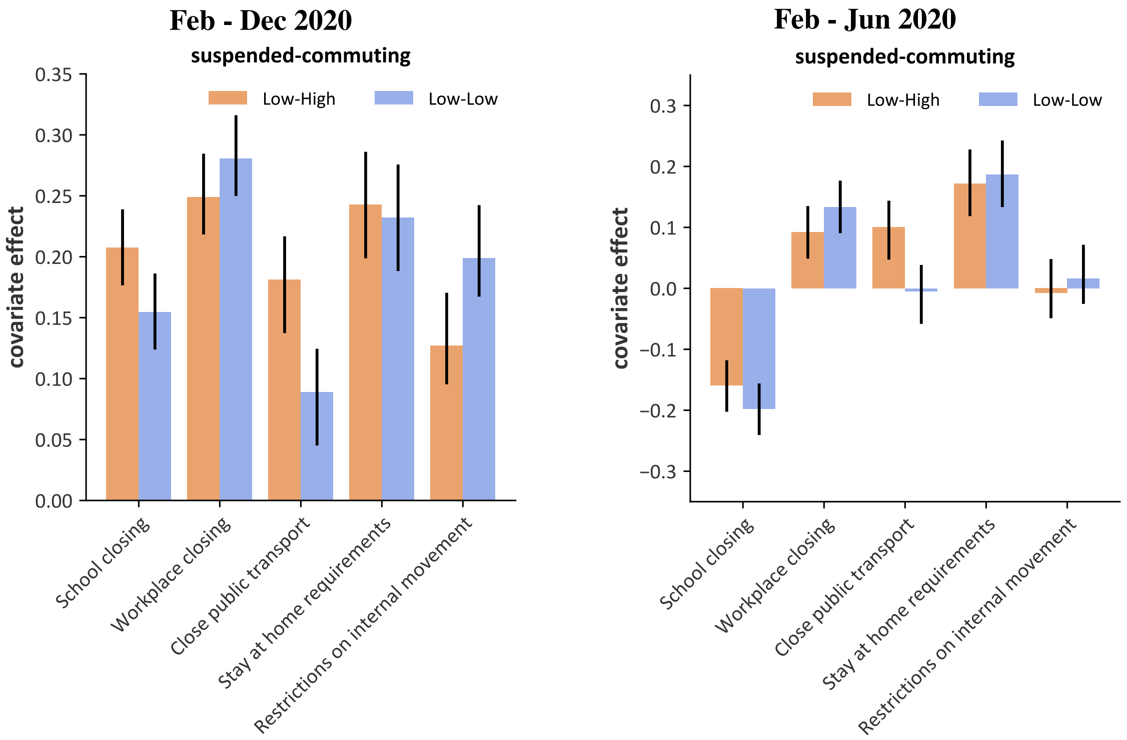

Figure SI 32. *Parameter estimates starting from February 11, 2020, until the end of the year*. Parameters are estimated using the Generalized Least Squares (GLS) method over a period starting from February 11, 2020, until the end of the year. Right: *Parameter estimates for only the early-pandemic period*. Parameters are estimated using the GLS method over a period starting from February 11, 2020, until June 10, 2020.

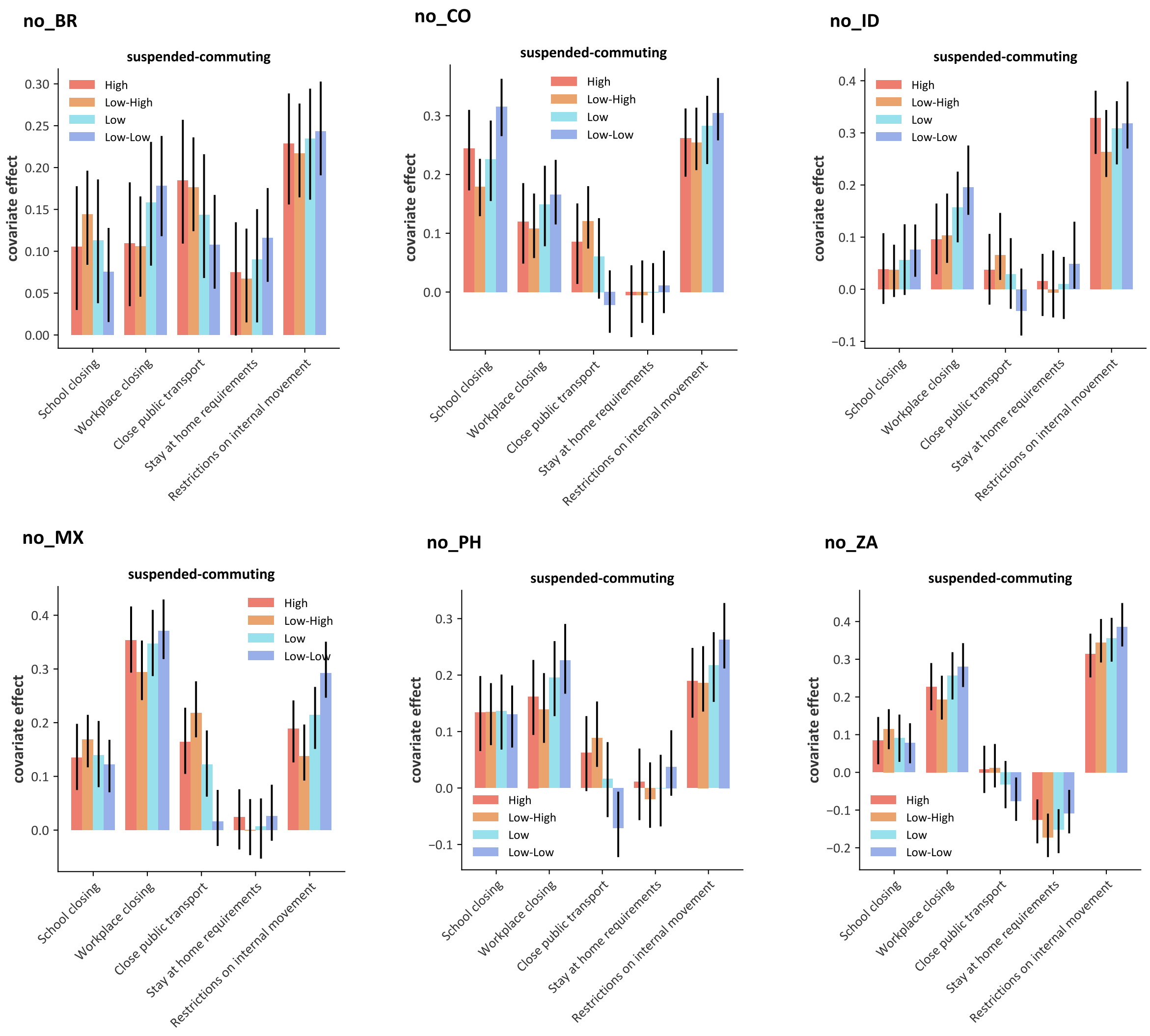

Figure SI 33. *Cross-validating the higher impact of transportation closure on low-wealth individuals working in high-wealth neighborhoods with respect to low-wealth individuals working in low-wealth neighborhoods.*

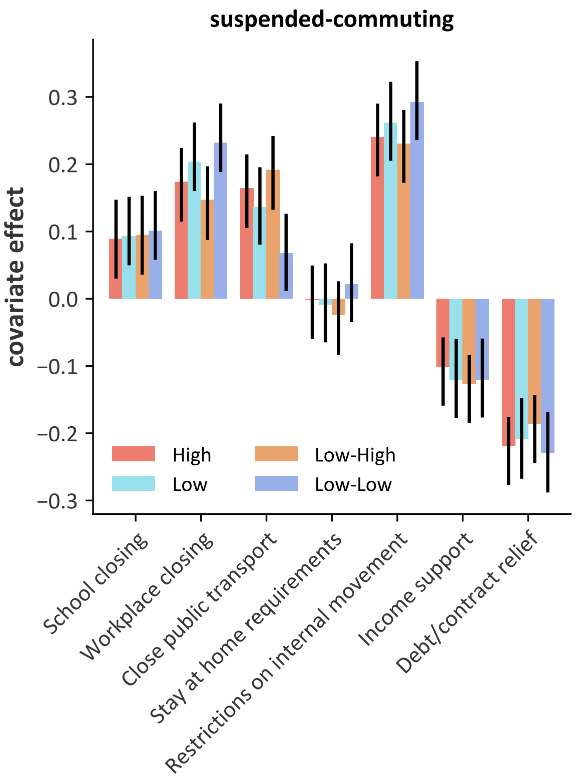

Figure SI 34. *Modeling with additional policy indices.* Results from modeling the fraction of individuals suspending their commuting patterns with two additional policy indices: income support policy index and debt/contract relief policy index.

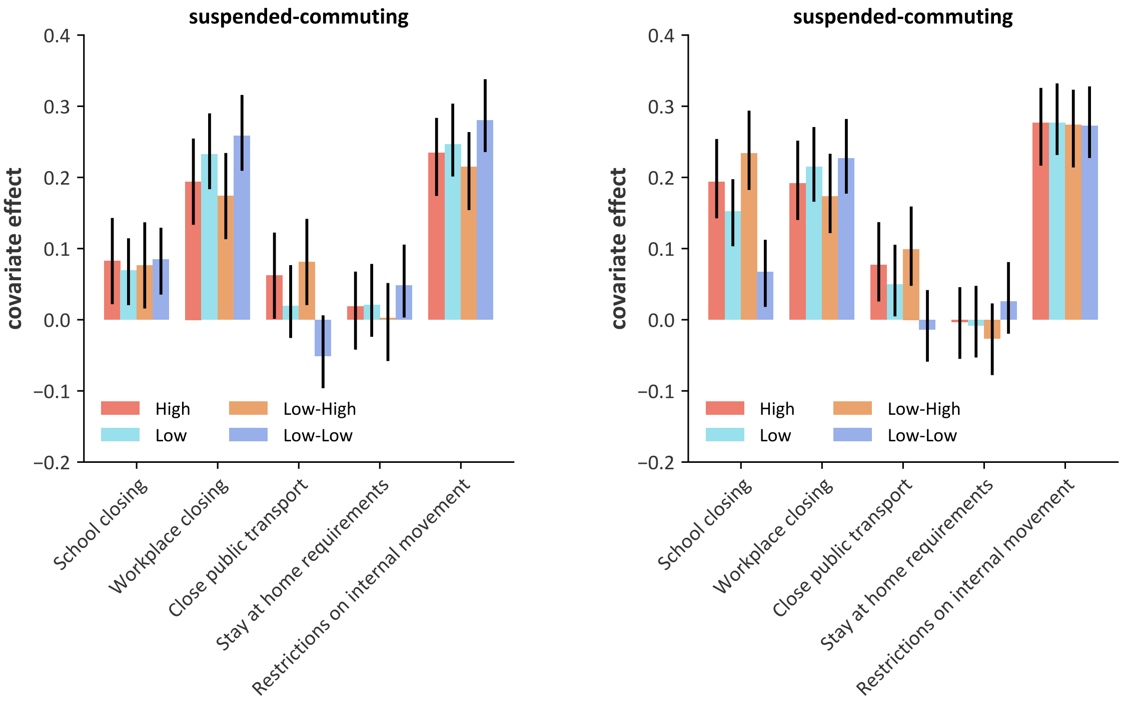

Figure SI 35. *Modeling with only one epidemiological indicator*. Left: parameter values obtained by including as epidemiological indicator only the daily global incidence of cases. Right: parameter values obtained by including as epidemiological indicator only the daily local (for each different country) incidence of cases.

SI 13. MULTI-DIMENSIONAL ANALYSIS OF BEHAVIORAL RESPONSES

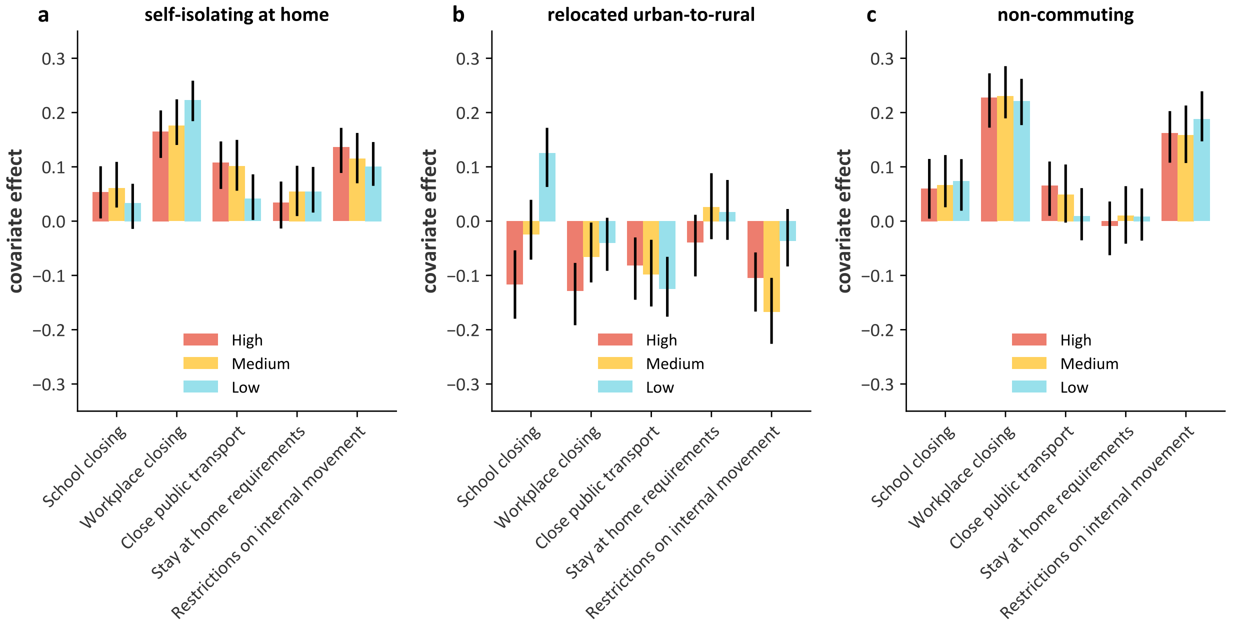

Figure SI 36. Modeling behavioral response to case incidence and containment policies. Panel a-c show the average effect of every policy covariate for the three mobility indicators: self-isolating at home (a), people who stopped commuting (b), and daily migratory flow from urban to rural areas (c).

Self-isolating at home, relocating from urban to rural areas, and suspending commuting activities are important indicators of how deeply an individual changed behavior over time. We use each of these three behavioral proxies separately as target variables of a multivariate panel regression model. Each indicator is studied in terms of both epidemiological conditions and enacted containment policies' covariates. Focusing on the different enacted policies and their stringency over time we include school closure, workplace closure, public transport closure, stay-at-home requirements, and internal movement restriction policies.

We find that all the containment policies are positively correlated with an increase in the fraction of individuals isolating at home as well as with a reduction in the fraction of individuals commuting to their workplace. In particular, the high-wealth group is associated on average with a higher (significant with an $\alpha = 0.05$) behavioral recovery to self-isolate when public transport closures are put in place and a lower propensity with respect to the low-wealth group (see Fig.SI 36a). Self-isolating patterns provide an important behavioral proxy, e.g., from an epidemiological and social perspective, but they have limited power in revealing the potential economic repercussions of containment measures on the job market and in turn on the economy of a country. With this in mind, we analyze, using the same statistical framework, the fraction of individuals who suspended their commuting patterns (trips between home and work location) over time. Thus, we focus only on individuals who were assigned a work location at least once over the study period.

Surprisingly, we find that the significant inter-group differences we found in self-isolation patterns become less relevant and not statistically significant (see Fig.SI 36c). Speculatively, this disparities reduction can be attributed to an inherently more similar mobility behavior of individuals who are commuting: if we focus only on individuals who are commuting (as long as they are commuting), a part of their daily mobility behavior can be explained in terms of commuting patterns, thus limiting behavioral changes to either the off-commuting pattern or the commuting pattern interruptions dimensions. In this respect, we focus on the latter dimension, i.e. the external margin of commuting behavior, as a proxy for job interruptions to capture macro disparities in the work-related group-behavior response to policy implementation.

Significant differences are found also in relocating patterns. However, these results are not robust and parameters' values and signs change depending on the robustness tests performed. These results were tested using the same robustness tests as those used for the model of "suspension of commuting patterns". For this reason, we decided not to discuss in depth these findings.

A. Policy indices over time

To give a sense of how single-policy indices are behaving for each country over the entire study period, we report here their values and computation procedure following the definition in [8].

1. Description of policy indices computation

Each policy index score $I_j(t)$ for a given indicator j on any given day t is computed using the following parameters (for a more detailed description see [15]):

- The maximum value of the policy indicator, N_j (having different values based on the different policy indicators, ranging from 2 to 5);
- Whether the indicator has a flag ($F_j = 1$ if it has a flag variable, 0 otherwise)
- The recorded policy value on the ordinal scale ($v_j(t)$)
- The recorded binary flag for the indicator ($f_j(t)$)

The calculation index computation balance these component into a real-valued variable, following the equation:

$$I_j(t) = 100\frac{v_j(t) - 0.5(F_j - f_j(t))}{N_j} \tag{6}$$

This normalizes the different ordinal scales to produce a policy index score between 0 and 100, where each full point on the ordinal scale is equally spaced. For indicators with a flag variable, if the flag is recorded as 0 (indicating a geographically targeted policy, or in the case of $E1$, support only for informal sector workers), this is treated as a half-step between ordinal values.

If a government has no policy for a given indicator ($v_j(t) = 0$), the corresponding flag is blank or null in the database. For the purpose of calculating the index, this is equivalent to a policy index score of zero. Hence, $I_j(t) = 0$ if $v_j(t) = 0$.

Finally, we divide each index by 100 to limit their codomain between 0 and 1.

See Fig. SI 37, where we have depicted the following policy implementations:

- **C1**: The index records policies closing schools and universities;
- **C2**: The index records policies closing workplaces;
- **C5**: The index records policies closing public transport;
- **C6**: The index records orders to 'shelter-in-place' and to, otherwise, confine at home;
- **C7**: The index records policy restrictions on internal movement between cities/regions.

Policy implementations concerning the canceling of public events (C3), limiting public gatherings (C4), and limiting/controlling international travels (C8) are not included in the analysis as they have a limited impact on individual mobility behavior at the country scale. In particular, control of international travel is expected to have relevant impacts on international-scale mobility, however, we do not have records of international movements in the data used for this analysis.

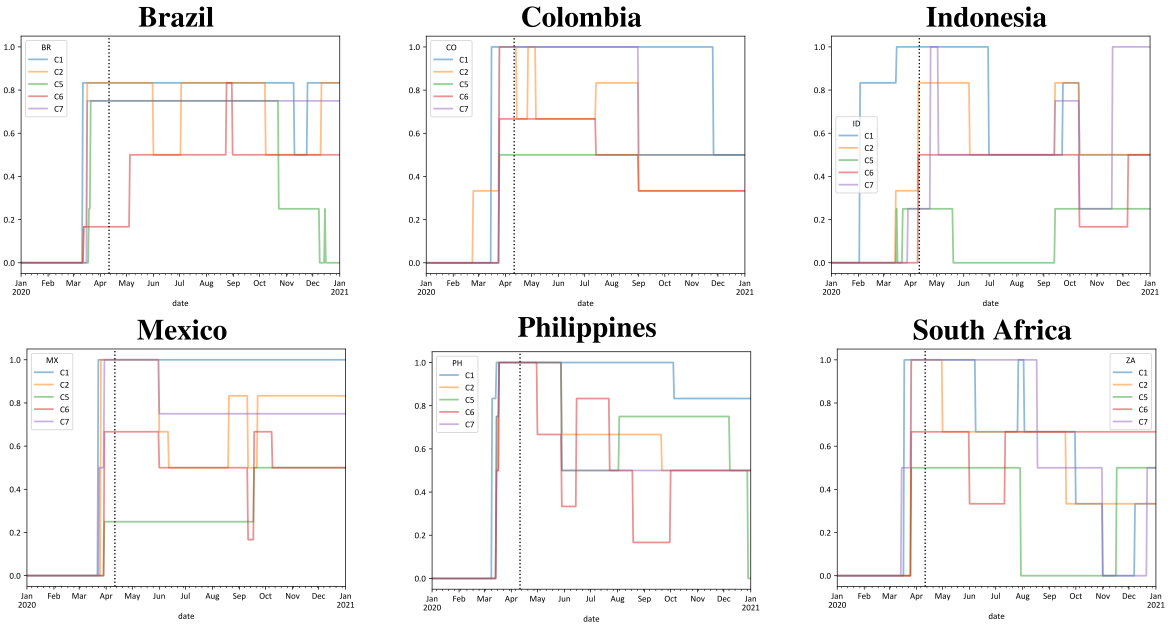

Figure SI 37. *Single-policy indices for containment policy type.* Each panel reports the index values over the entire period of our study. Colors represent the different single-policy indices. The vertical line represents the starting date that we used to perform the regression in the model presented in the main manuscript: April 11, 2020, namely one month after the pandemic declaration.

SI 14. CONTROLLING FOR GEOGRAPHIC AND WEALTH BIASES IN THE USER BASE

Figure SI. SI 38 shows for each of the countries the relation between the population residing within a specific administrative unit and the user population residing in the same administrative unit. In particular, the strong correlation between the two quantities shows the overall geographical representativity of the mobile phone user population in all countries. Moreover, Fig. SI. SI 39 reports, for each country, the distribution by wealth group of the ration between the user population and the administrative unit population. Across all countries, except for South Africa (ZA), we don't see a strong and systematic relation between the wealth groups and the values of the "user pop / admin pop" ratio.

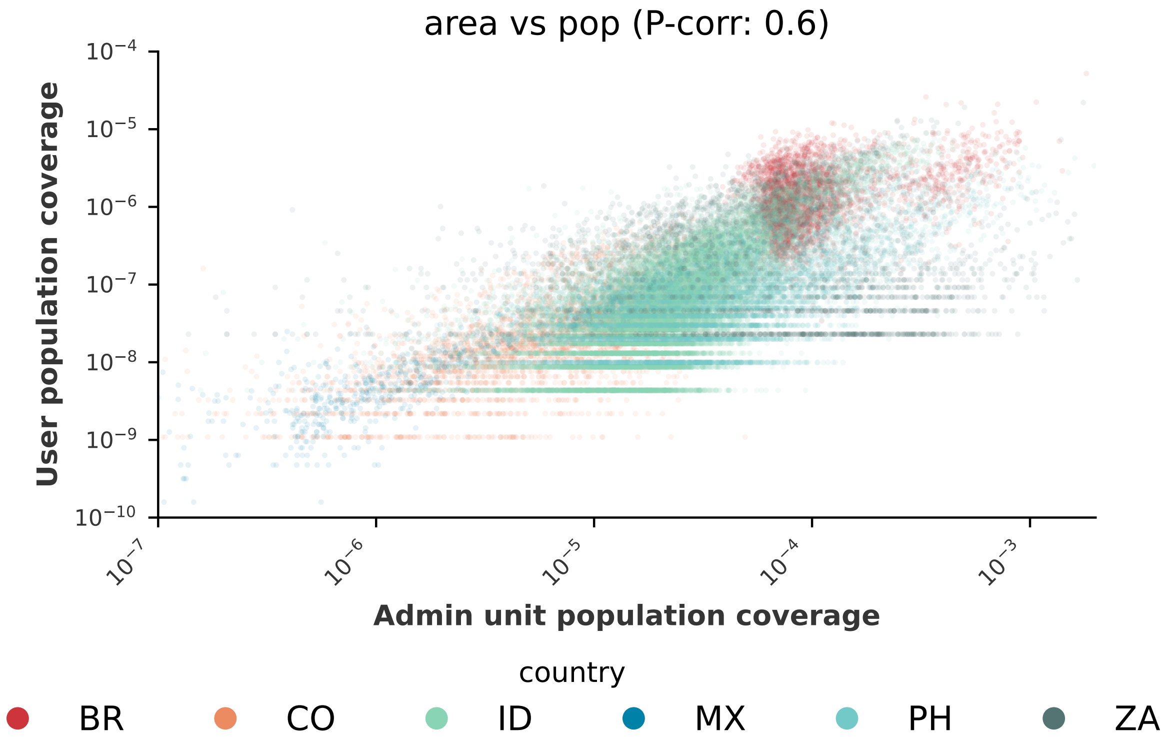

Figure SI 38. Comparing administrative units' population and user-base population. The shows the relation between an administrative unit population (reported as a fraction of the country population) and the user population residing in the specific administrative unit (reported as a fraction of the country population). Both axes are reported in log-scale. At the top of the figure, we report the average Pearson correlation coefficient across all countries.

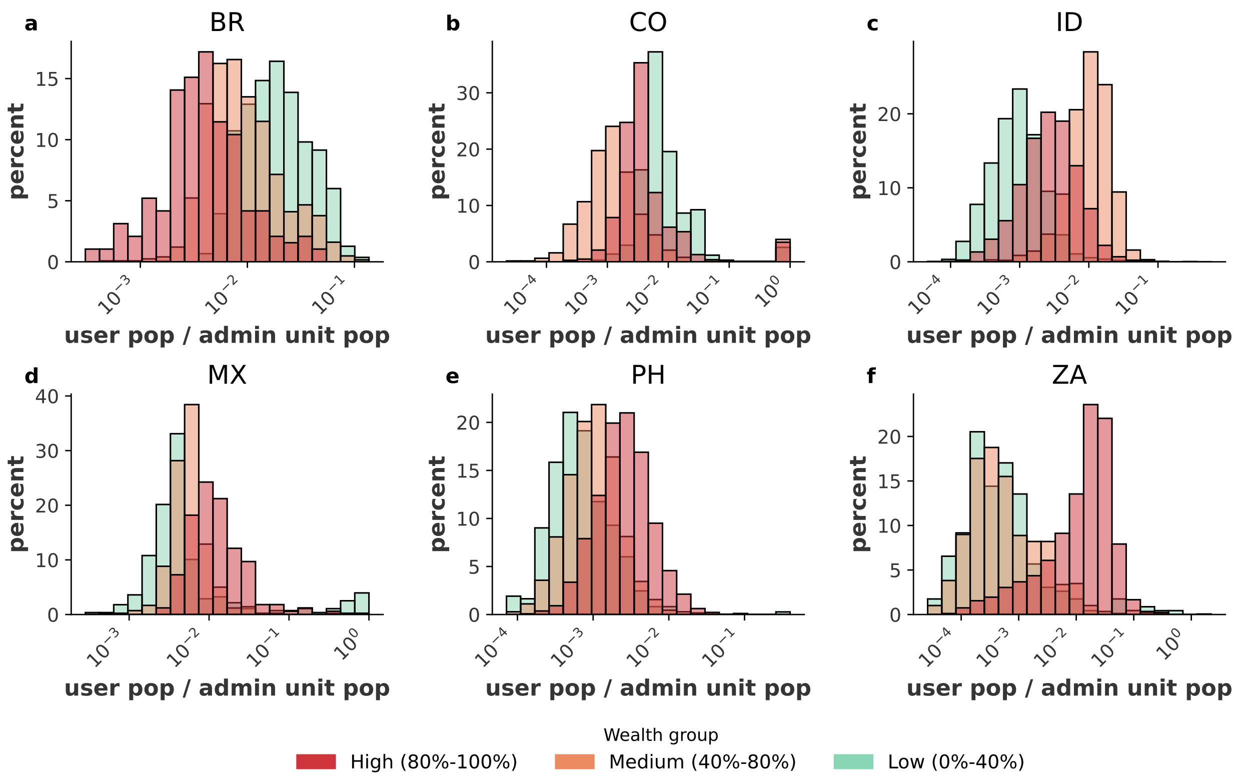

Figure SI 39. Distribution of the user population and administrative unit ratio by wealth group. Each of the panels shows the log-binned histogram of the administrative units, with the x-axis reporting the ratio between the user population residing in each administrative unit and the corresponding administrative unit population. The y-axis reports the percentage of each group's administrative units counted in each bin.

[1] Lauren Alexander, Shan Jiang, Mikel Murga, and Marta C González. Origin–destination trips by purpose and time of day inferred from mobile phone data. *Transportation research part c: emerging technologies*, 58:240–250, 2015.

[2] Ulf Aslak and Laura Alessandretti. Infostop: Scalable stop-location detection in multi-user mobility data, 2020.

[3] Hugo Barbosa, Fernando B de Lima-Neto, Alexandre Evsukoff, and Ronaldo Menezes. The effect of recency to human mobility. *EPJ Data Science*, 4(1):1–14, 2015.

[4] Peter A Burrough, Rachael McDonnell, Rachael A McDonnell, and Christopher D Lloyd. *Principles of geographical information systems*. Oxford university press, 2015.

[5] Serdar Çolak, Lauren P Alexander, Bernardo G Alvim, Shomik R Mehndiratta, and Marta C González. Analyzing cell phone location data for urban travel: current methods, limitations, and opportunities. *Transportation Research Record*, 2526(1):126–135, 2015.

[6] Balázs Cs Csáji, Arnaud Browet, Vincent A Traag, Jean-Charles Delvenne, Etienne Huens, Paul Van Dooren, Zbigniew Smoreda, and Vincent D Blondel. Exploring the mobility of mobile phone users. *Physica A: statistical mechanics and its applications*, 392(6):1459–1473, 2013.

[7] Martin Ester, Hans-Peter Kriegel, Jörg Sander, Xiaowei Xu, et al. A density-based algorithm for discovering clusters in large spatial databases with noise. In *KDD*, volume 96, pages 226–231, 1996.

[8] Thomas Hale, Samuel Webster, Petherick Anna, Phillips Toby, and Kira Beatriz. Oxford covid-19 government response tracker. *Blavatnik school of government working paper*, 31:2020–11, 2020.

[9] Ramaswamy Hariharan and Kentaro Toyama. Project lachesis: parsing and modeling location histories. In *International Conference on Geographic Information Science*, pages 106–124. Springer, 2004.

[10] Gareth James, Daniela Witten, Trevor Hastie, Robert Tibshirani, et al. *An introduction to statistical learning*, volume 112. Springer, 2013.

[11] Zhenlong Li, Huan Ning, Fengrui Jing, and M Naser Lessani. Understanding the bias of mobile location data across spatial scales and over time: a comprehensive analysis of safegraph data in the united states. *Plos one*, 19(1):e0294430, 2024.

[12] Luca Pappalardo, Leo Ferres, Manuel Sacasa, Ciro Cattuto, and Loreto Bravo. An individual-level ground truth dataset for home location detection. *arXiv preprint arXiv:2010.08814*, 2010(08814):1–20, 2020.

[13] Chaoming Song, Tal Koren, Pu Wang, and Albert-László Barabási. Modelling the scaling properties of human mobility. *Nature physics*, 6(10):818–823, 2010.

[14] Oxford COVID-19 Government Response Tracker. Oxcgrt/covid-policy-tracker: Systematic dataset of covid-19 policy, from oxford university. Accessed on 2023-02-24.

[15] Oxford Covid-19 Government Response Tracker. covid-policy-tracker/documentation/index_methodology.md at master · oxcgrt/covid-policy-tracker · github, 2022.

[16] Uber. h3/docs at github master - uber/h3, Accessed on 2020-10-14.

[17] Veraset. Veraset datasheet movement - documentation, 2021. Accessed on 2021-11-19.

[18] Veraset. Veraset movement data – veraset, 2021. Accessed on 2021-02-17.

[19] Jia Yu, Jinxuan Wu, and Mohamed Sarwat. Geospark: A cluster computing framework for processing large-scale spatial data. In *Proceedings of the 23rd SIGSPATIAL International Conference on Advances in Geographic Information Systems*, pages 1–4, 2015.
